# Epidemiological analysis and temporal trends of interstitial lung diseases in global, Chinese, and Belt and Road Initiative countries: 1990–2021

**DOI:** 10.3389/fmed.2025.1620714

**Published:** 2025-08-29

**Authors:** Tianyu Si, Xiawei Shi, Jiayi Ma, Junchao Yang

**Affiliations:** ^1^Zhejiang Chinese Medical University, Hangzhou, China; ^2^The First Affiliated Hospital of Zhejiang Chinese Medical University, Hangzhou, China

**Keywords:** Belt and Road Initiative countries, the global burden of disease, interstitial lung disease, epidemiology, disability-adjusted life years

## Abstract

**Background:**

This study aims to comprehensively evaluate the historical, current, and projected burden of interstitial lung diseases (ILD) across global populations, China, and the Belt and Road Initiative (BRI) countries. Additionally, exploring cross-national inequalities across socio-demographic index (SDI).

**Methods:**

From the 2021 Global Burden of Disease (GBD) database, we selected data on interstitial lung diseases (ILD) for global populations, China, and BRI countries. We analyzed the changes in the burden of ILDs according to year, sex, location, age, and SDI, and used the estimated annual percentage change (EAPC) to estimate the trends of the disease burden. Time trends were evaluated using Joinpoint analysis, while health disparities were assessed with the inequality slope index and concentration index. Additionally, the autoregressive integrated moving average (ARIMA) model was employed to forecast the future trends.

**Results:**

From 1990 to 2021, the global age-standardized incidence rate (ASIR) of interstitial lung diseases and sarcoidosis (ILD) increased from 3.77 per 100,000 (95% UI: 3.27, 4.28) to 4.55 per 100,000 (95% UI: 4.06, 5.04), with an EAPC of 0.73 (95% CI: 0.63, 0.82). Both the age-standardized mortality rate (ASMR) and the age-standardized disability-adjusted life year (DALY) rate (ASDR) also showed an increase. In 2021, China’s ASIR was 2.32 per 100,000 (95% UI: 2.03, 2.65), ASMR was 0.39 per 100,000 (95% UI: 0.24, 0.53), and ASDR was 10.82 per 100,000 (95% UI: 7.70, 13.97). When it comes to rankings among BRI countries, China ranked 49.36% for ASIR, 17.95% for ASMR, and 17.31% for ASDR, from lowest to highest. Countries with higher SDI along the BRI countries had a faster annual average growth rate in ILD incidence, and the inequality in ILD between high SDI and low SDI countries was gradually increasing, albeit to a smaller extent. Predicting the trend of ASDR by 2031, it showed a global downward trend, while it showed an upward trend in Chinese.

**Conclusion:**

The ILD burden of BRI countries varies by region, gender, and time factors, and the unbalanced development of their regions exacerbates the imbalance of burden. Therefore, it is necessary to pay attention to and strengthen cooperation in the health field of BRI countries and promote the rational allocation of medical resources to help realize the construction of a community of human destiny.

## 1 Introduction

Interstitial lung disease (ILD) is a group of heterogeneous pulmonary parenchymal diseases that encompass over 200 conditions, characterized by inflammation or fibrosis in the lung interstitium, alveolar walls, and alveolar spaces, leading to impaired gas exchange, respiratory symptoms, and lung function decline (1). Sarcoidosis is an organ-affected granulomatous disease of unknown cause, most commonly affecting the lungs and causing ILD, with granuloma formation mechanisms often related to genetic and environmental factors (2, 3). Idiopathic pulmonary fibrosis (IPF) is the most severe and common form of ILD, characterized by persistent and often worsening fibrosis, with pirfenidone and nintedanib, widely used currently, only slowing the decline in forced vital capacity but not improving survival rates (4). Due to the irreversible lung damage caused by ILD, with over 20 million cases of ILD-related diseases globally in 2019 (5), and significant regional heterogeneity (4, 6), it imposes a heavy social and economic burden on countries, with median annual direct costs equivalent to 50% of per capita GDP (7).

In 2013, China first proposed the “Belt and Road” Initiative (BRI), which includes the “Silk Road Economic Belt” and the “21st Century Maritime Silk Road.” As of April 2025, 156 countries on six continents have responded to the BRI and reached cooperation and co-construction agreements (8). The cooperating countries and regions occupy a vast space on the planet and are made up of regions with different levels of socio-economic development (9). BRI has promoted the improvement of medical and health infrastructure in countries along the Belt and Road through infrastructure investment, and the Chinese government has launched the “Health Silk Road” initiative to strengthen global health cooperation and the flow of medical resources.

The development of the health sector serves as one of the intrinsic driving forces for the BRI and is directly related to the national and regional sustainable development goals (10). However, China and its member countries are currently facing varying degrees of ILD threats. A comparable and comprehensive analysis and assessment of ILD incidence rates, mortality rates, disease burden, and long-term trends are crucial, which can help in the formulation of prevention strategies and the allocation of resources for relevant countries and even globally.

## 2 Materials and methods

### 2.1 Data source

This study utilized the publicly available data from the 2021 GBD study, which was obtained through the Global Health Data Exchange (GHDx).^1^ Our study utilized ILD data from the GBD database for global, 21 GBD regions, and 156 BRI countries during the period of 1990–2021. Additionally, these countries and regions were further divided into five areas based on the Socioeconomic Index (SDI), including low, low-middle, middle, high-middle, and high categories. We conducted age-standardization using various epidemiological data, such as incidence, mortality, and Disability-Adjusted Life Years (DALYs). The analysis of publicly available de-identified data in our study did not involve human or animal subjects, and therefore, it did not require ethical approval.

### 2.2 Joinpoint analysis

Use Joinpoint software (Version 5.2.0, Statistical Research and Applications Branch, National Cancer Institute^2^) to evaluate the GBD data, analyzing the trends of age-standardized incidence rates (ASIR), age-standardized mortality rates (ASMR), and age-standardized DALYs rates (ASDR) for global and Chinese ILD. Joinpoint regression analysis models trends as a series of connected linear segments, each representing a period of constant percentage change. The points where segments connect are called “joinpoints,” indicating statistically significant changes in trends (11).

### 2.3 Cross-national social relevance and inequality analysis

The Spearman rank correlation analysis method was used to evaluate the association between SDI and ILD incidence rates in BRI countries in 2021, as well as the EAPC of ILD incidence rates from 1990 to 2021. Scatter plots were generated to visualize these correlations, including the Spearman rank correlation coefficient (ρ) and the corresponding *p*-value to quantify the strength and statistical significance of the relationship (12).

The inequality slope index and health inequality concentration index were used to assess the unequal distribution of ILD burden across BRI countries. These indices represent standard measures of absolute and relative gradient inequality, respectively. By regressing the ASDR due to ILD in BRI countries against the relative position scale of SDI, the inequality index slope was calculated. This scale was defined by the midpoint of the cumulative range of the population ranked by SDI. The health inequality concentration index was calculated by numerically integrating the area under the Lorenz concentration curve, which was fitted using the cumulative fraction of DALYs and the cumulative relative distribution of the population ranked by SDI (13, 14). Some analyses suggest that an absolute value of 0.2–0.3 represents a relatively high level of relative inequality (15).

### 2.4 ARIMA (auto-regressive integrated moving average) model

Based on the historical data of ASDR, the ARIMA model is used to make predictions for the next 10 years globally and in China. The ARIMA model includes three main components: auto-regression (AR), moving average (MA), and integration (I). Its standard notation is ARIMA (p, d, q), where “p” represents the order of auto-regression, “d” represents the order of differencing, and “q” represents the order of moving average (16). The basic principle of the ARIMA model is to conceptualize the data generated by the target variable over time as a random sequence, and to predict future values by utilizing the relationships between historical values and current values in the sequence (17). The auto.arima() function is mainly used for ARIMA models with different time series data, searching and determining the optimal model based on the constraints provided (18, 19). In this study, the optimal model was established using auto.arima() according to the Akaike Information Criterion (AIC) values, and the normality of the model residuals was verified by the residual QQ plot, autocorrelation function (ACF) plot and partial autocorrelation function (PACF) plot. The Ljung-Box test for white noise was used to test whether the residuals were serially correlated.

### 2.5 Statistical analysis

All analyses and visualizations in this study were analyzed using R software (version 4.4.2) to elucidate the burden of disease in global, Chinese and BRI countries.

## 3 Result

### 3.1 The incidence, mortality, and DALY rates of interstitial lung disease from 1990 to 2021 in global, regional, and BRI countries trends

#### 3.1.1 Incidence

**FIGURE 1:**
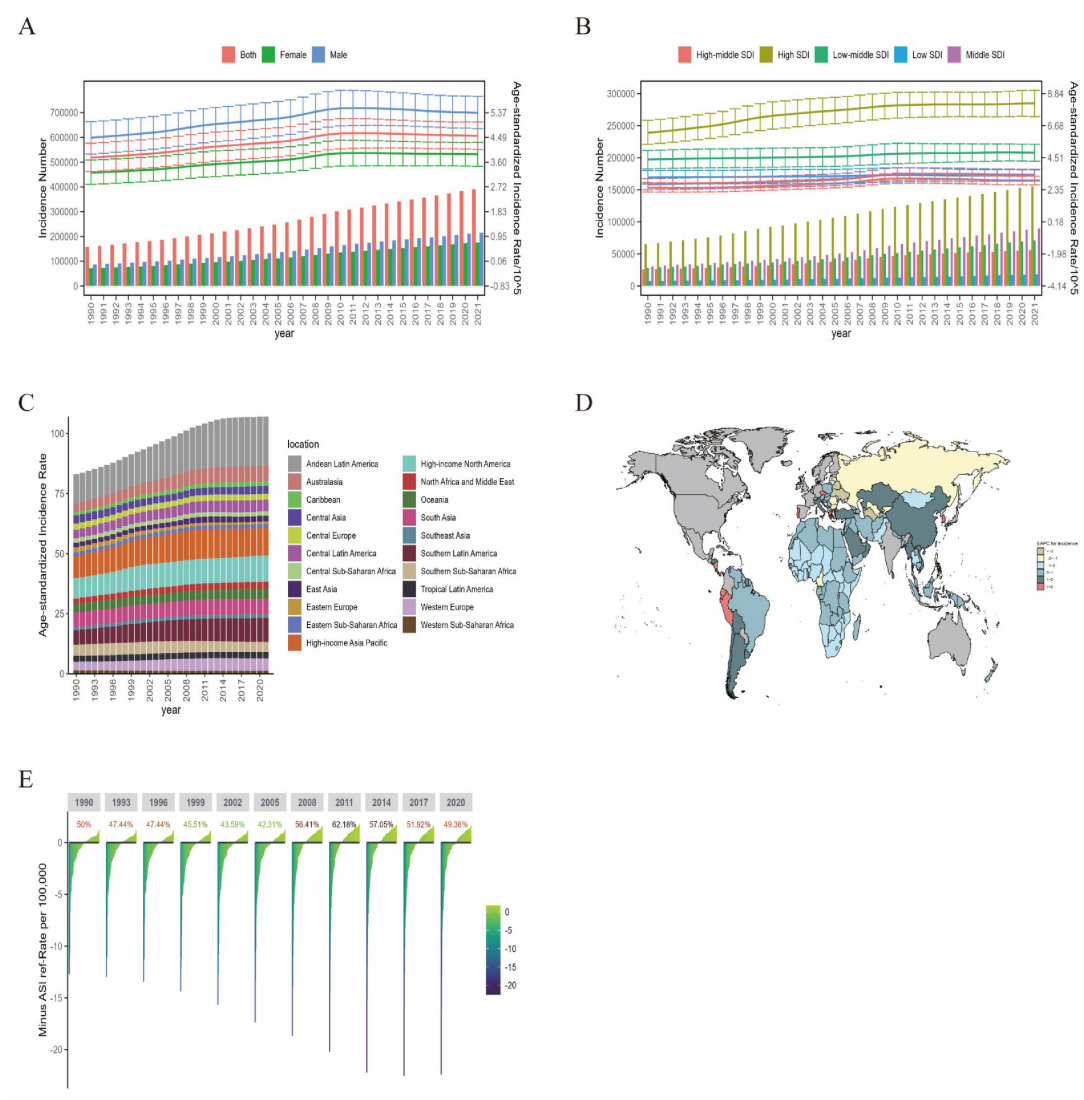
The incidence burden of ILD in global, regional, and BRI countries from 1990 to 2021. **(A)** Global number of incidence cases and ASIR; **(B)** global number of incidence cases and ASIR by SDI regions; **(C)** global number of ASIR by 21 GBD regions; **(D)** EAPC of ILD incidence in BRI countries (1990–2021); **(E)** the ASIR reference ranking of China among the BRI countries.

According to GBD 2021, in 2021 it is estimated that there were 390.27 [95% uncertainty interval (UI): 346.39, 433.40] thousand cases of ILD. The ASIR in 2021 was 4.55 per 100,000 (95% UI: 4.06, 5.04), an increase of 20.7% from 1990, with an Estimated Annual Percentage Change (EAPC) of 0.73 (95% CI: 0.63, 0.82). By gender, the ASIR increased by 19.9% for males and 20.4% for females, with EAPC being roughly the same (Figure 1A and Supplementary Table 1).

From different SDI regions, High SDI regions had the most IDL cases and the highest ASIR in 2021, at 155.24 (95% UI: 137.46, 174.12) thousand cases and 8.19 per 100,000 (95% UI: 7.29, 9.07). Low SDI regions had the least IDL cases in 2021 (Figure 1B), with the smallest EAPC of 0.17 (95% CI: 0.13, 0.19). Among the 21 global regions in 2021, the highest ASIR was in Andean Latin America, High-income Asia Pacific, and High-income North America, with Andean Latin America being the highest at 20.47 per 100,000 (95% UI: 19.15, 21.69); the lowest regions were Eastern Sub-Saharan Africa, Western Sub-Saharan Africa, and Eastern Europe, with Eastern Europe being the lowest at 1.04 per 100,000 (95% UI: 0.89, 1.21) (Figure 1C and Supplementary Table 1).

The analysis of BRI countries shows that ASIR has not changed significantly from 1990 to 2021, among which the highest countries in 1990 are Peru, Brunei Darussalam, Bolivia (Plurinational State of), and in 2021, the highest countries are Peru, Bolivia (Plurinational State of), Chile, Ecuador, Maldive, and Brunei Darussalam, each with more than 10 cases per 100,000 population (Figure 1D). China’s ASIR went from 1.92 per 100,000 in 1990 (95% UI: 1.62, 2.26) to 2.32 per 100,000 in 2021 (95% UI: 2.03, 2.65), with an EAPC of 1.11 (95% CI: 0.83, 1.38) (Supplementary Table 1). China’s ASIR ranking among BRI countries was 49.36% in 2021, which has decreased since 2011 (Figure 1E).

#### 3.1.2 Mortality

**FIGURE 2:**
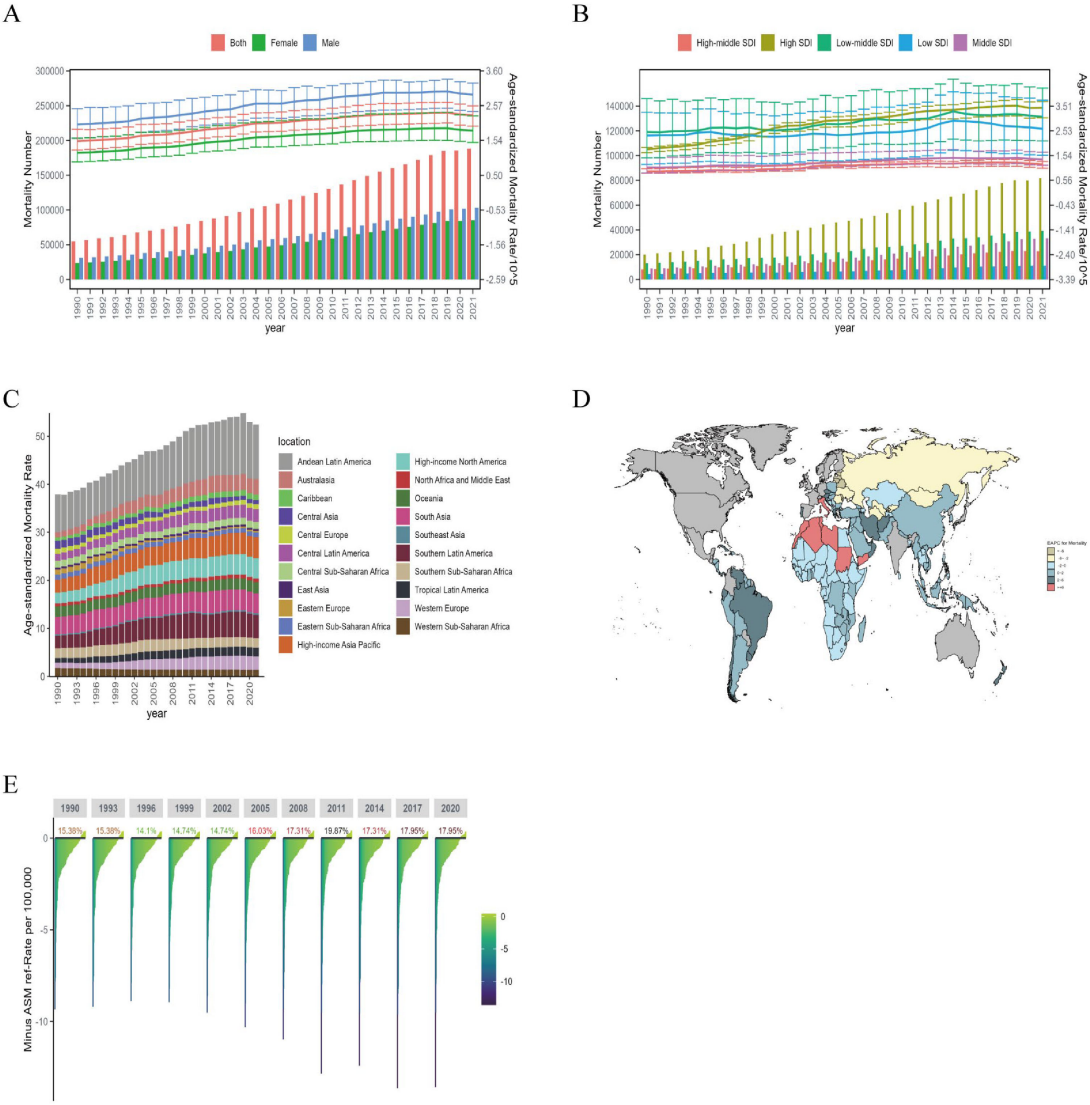
The mortality burden of ILD in global, regional, and BRI countries from 1990 to 2021. **(A)** Global number of mortality cases and ASMR; **(B)** global number of mortality cases and ASMR by SDI regions; **(C)** global number of ASMR by 21 GBD regions; **(D)** EAPC of ILD mortality in BRI countries (1990–2021); **(E)** the ASMR reference ranking of China among the BRI countries.

Globally, in 2021, there were approximately 188,220 [95% UI: 161,410, 212,250] deaths from ILD cases, an increase of 242.4% increase from 1990 (Figure 2A), with ASMR rising to 2.28 per 100,000 (95% UI: 1.96, 2.56), and EAPC at 1.55 (95% CI: 1.41, 1.70). By gender, in 2021, the ASMR was 2.90 per 100,000 (95% UI: 2.40, 3.24) people for males, significantly higher than the 1.83 per 100,000 (95% UI: 1.48, 2.27) for females (Figure 2A), while the EAPC was slightly higher for females (Supplementary Table 2).

By different SDI regions, it can be seen that in 2021, High SDI regions had the highest number of IDL deaths and ASMR, with 81.73 (95% UI: 71.24, 88.09) thousand cases and 3.44 per 100,000 (95% UI: 3.05, 3.69), respectively, with the highest EAPC of 2.30 (95% CI: 2.05, 2.56) from 1990 to 2021. Other SDI regions saw an increase in ASMR, with the lowest EAPC in Low SDI regions at 0.63 (95% CI: 0.45, 0.82) (Figure 2B). Among the 21 global regions, Andean Latin America had the highest ASMR in 2021 at 11.37 per 100,000 (95% UI: 8.69, 14.33). The regions with the highest EAPC were Western Europe at 3.74 (95% CI: 3.38, 4.11) and Australasia at 3.55 (95% CI: 2.98, 4.11), while the lowest EAPC was in Eastern Europe and Central Asia at −5.47 (95% CI: −6.40, −4.54) and −2.05 (95% CI: −2.51, −1.60) (Figure 2C and Supplementary Table 2).

After analyzing the BRI countries, it can be observed that the overall ASMR has been increasing from 1990 to 2021, with Peru being the country with the highest ASMR in both 1990 and 2021; the countries with the highest EPAC are Italy, Libya, and Morocco, while the countries with the lowest EPAC are the Republic of Moldova, Latvia, Estonia, and Lithuania (Figure 2D). In 1990, the number of deaths due to ILD in China was 2.96 (95% UI: 2.19, 4.69) thousand cases, while in 2021 it was 7.67 (95% UI: 4.64, 10.37) people, and the ASMR decreased from 0.41 per 100,000 (95% UI: 0.31, 0.67) to 0.39 per 100,000 (95% UI: 0.24, 0.53) (Supplementary Table 2). In 2021, China ranked 17.95% in BRI countries in terms of ASMR (Figure 2E).

#### 3.1.3 DALYs

**FIGURE 3:**
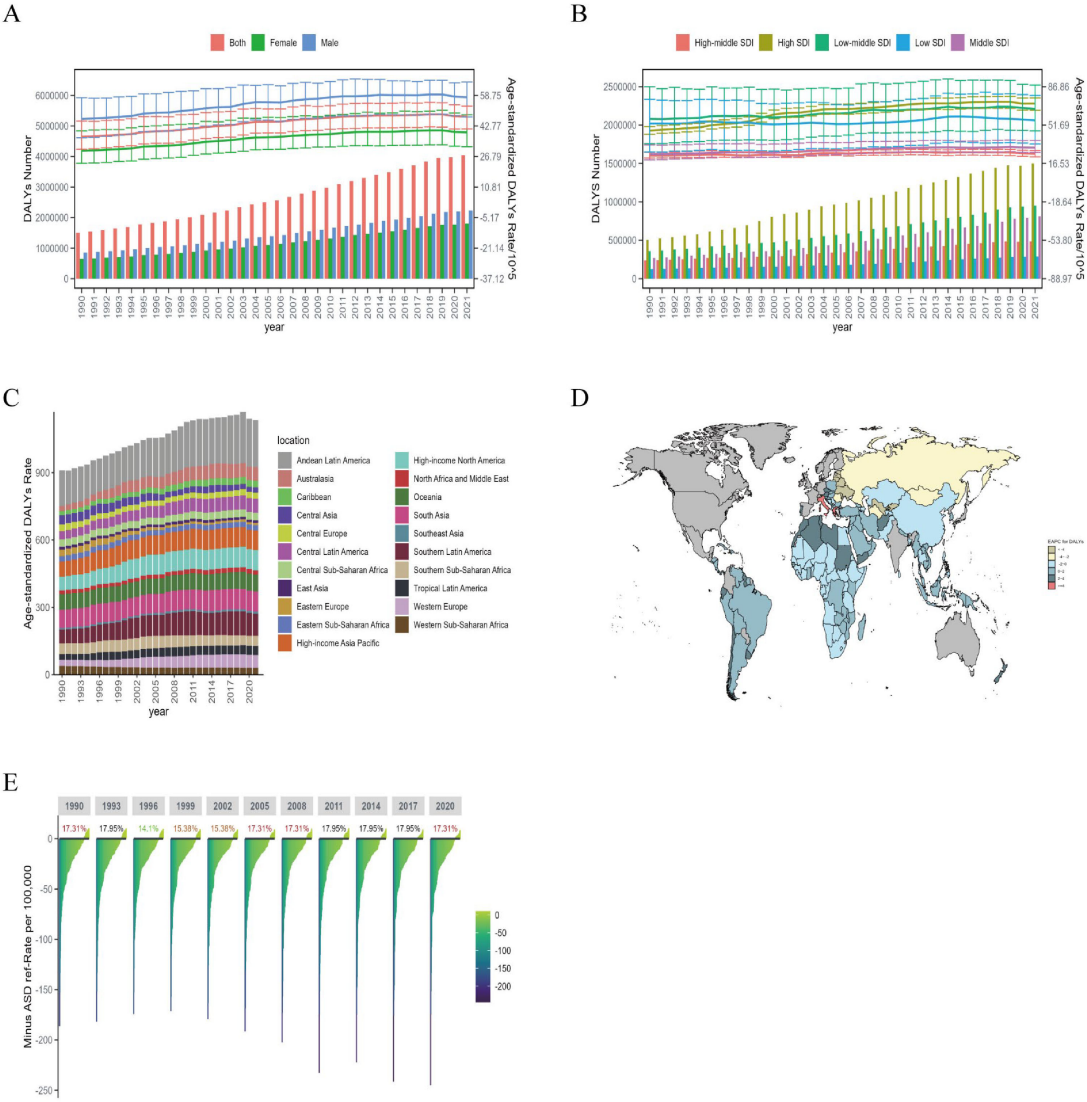
The DALYs burden of ILD in global, regional, and BRI countries from 1990 to 2021. **(A)** Global number of DALYs cases and ASDR; **(B)** global number of DALYs cases and ASDR by SDI regions; **(C)** global number of ASDR by 21 GBD regions; **(D)** EAPC of ILD DALYs in BRI countries (1990–2021); **(E)** the ASDR reference ranking of China among the BRI countries.

Globally, DALYs due to ILD rose 169.3% from 1990 to 2021 to 4,042.15 [95% UI 3489.80, 4,516.88] thousand cases, while ASDR rose to 47.62 per 100,000 (95% UI: 41.26, 53.17). In terms of gender, it can be seen that the trend of ASDR is roughly the same as that of ASMR, with ASDR higher for men than women in 2021, while EAPC 1.07 (95% CI: 0.96, 1.17) for women is higher than 0.85 (95% CI: 0.76, 0.95) for men (Figure 3A and Supplementary Table 3).

Analysis shows that in 2021, ASDR in different SDI regions, except for High-middle SDI and Middle SDI regions, were at a high level. The highest EAPC region from 1990 to 2021 was High SDI at 1.54 (95% CI: 1.33, 1.75) (Figure 3B). Among the 21 global regions, Andean Latin America had the highest ASDR at 209.34 per 100,000 (95% UI: 165.81, 257.67) in 2021. The regions with the highest EAPC were Australasia at 3.10 (95% CI: 2.54, 3.66) and Western Europe at 3.00 (95% CI: 2.65, 3.35); the regions with the lowest EAPC were Eastern Europe and Central Asia at −4.56 (95% CI: −5.26, −3.84) and −1.98 (95% CI: −2.40, −1.57), respectively (Figure 3C and Supplementary Table 3).

Among the 156 BRI countries, the countries with the highest ASDR are the same as those with the highest ASMR, namely Peru, Bolivia (Plurinational State of), and Chile, with more than 150 cases per 100,000 people; the countries with the lowest ASDR are the Republic of Moldova, Iran (Islamic Republic of), and the Philippines, with fewer than 4 cases per 100,000 people. The countries with the highest EAPC are Italy and Greece, while the lowest are Latvia, Estonia, and Lithuania (Figure 3D). China’s ASDR decreased from 12.55 (95% UI: 9.49, 18.56) per 100,000 people in 1990 to 10.82 per 100,000 (95% UI: 7.70, 13.97) people in 2021, with an EAPC of −0.23 (95% CI: −0.38, −0.08) (Supplementary Table 3). In 2021, China ranked 17.31% among BRI countries in terms of ASDR (Figure 3E).

### 3.2 The burden of disease of ILD in different age groups in China from 1990 to 2021

**FIGURE 4:**
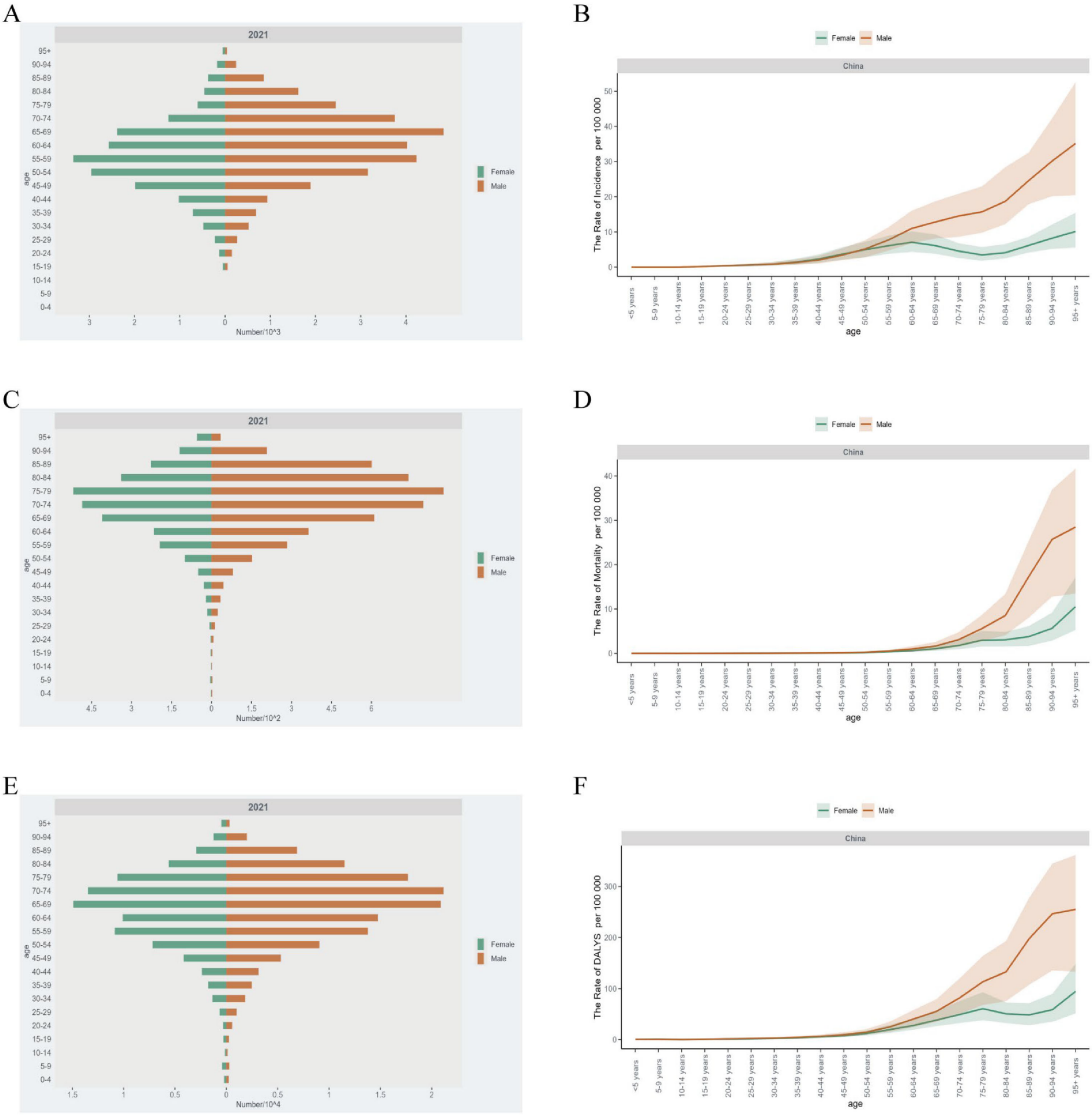
The burden of ILD in different age groups in China in 2021. **(A)** The number of incidence cases and **(B)** ASIR; **(C)** the number of mortality cases and **(D)** ASMR; **(E)** the number of DALYs cases and **(F)** ASDR.

Figure 4 shows the incidence, mortality, number of DALYs, and rates of ILD in different age groups in China in 2021. The incidence of ILD in China initially began to increase in the 35–39 age group. The incidence in males increased until the 95+ -years-old group, while in females it increased to the point where it began to decline after the 60–64 age group and increased again until the end in the 75–79 age group (Figure 4A). Mortality increases from the age group of 60 to 64 years (Figure 4C). Compared with mortality, the DALYs rate was 2 years earlier in the age group, starting in the 50–54 age group (Figure 4E). The incidence of ILD increases rapidly in individuals after the age of 45 years and peaks between the ages of 50 and 75 years. A similar trend has been observed in terms of DALYs and the number of deaths, but the peak is delayed by 1 or 2 age groups compared to incidence. The incidence and mortality of DALYs were higher in males than in females (Figures 4B–D).

In 2021, ILD incidence, mortality and DALYs rate in China reached the highest value in the 95 + age group, and the incidence rate EAPC in this group also reached the highest value of 3.32 (95% CI: 2.76, 3.89). The highest EAPC values for mortality and DALYs rates from 1990 to 2021 were in the 85–89 age group. EAPC of incidence rate, mortality and DALYs rate before 60 years old are all negative value, except that EAPC of incidence rate of 15–35 years old is positive (Supplementary Table 4).

### 3.3 Joinpoint regression analysis

**FIGURE 5:**
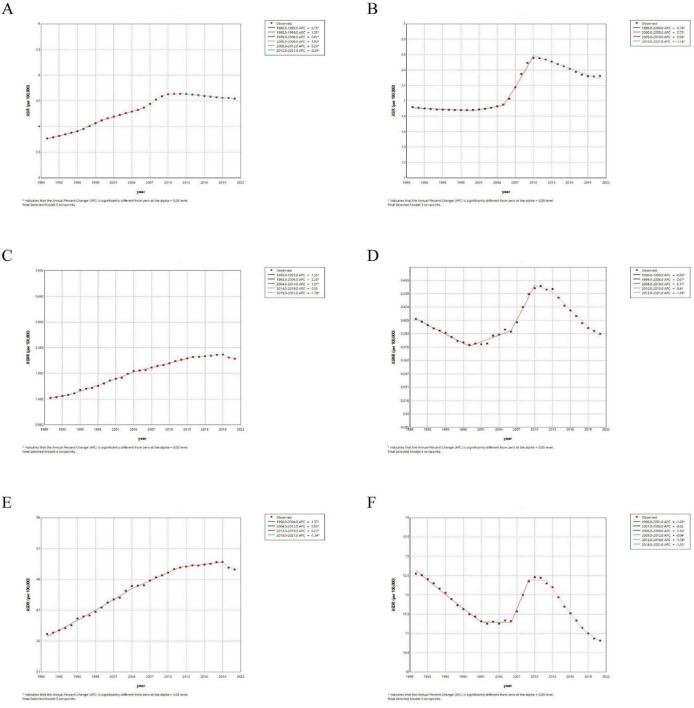
Joinpoint regression analysis of ILD from 1990 to 2021. The ASIR **(A)**, ASMR **(C)** and ASDR **(E)** in global; the ASIR **(B)**, ASMR **(D)** and ASDR **(F)** in China.

Figure 5 shows the Joinpoint regression analysis of ASIR, ASMR, and ASDR for global and Chinese ILD from 1990 to 2021. During this period, the global ASIR showed an increasing trend until 2012, followed by a slow declining trend until 2021, with an annual percentage change (APC) of −0.25 (95% CI: −0.28 ∼−0.23) (Figure 5A). Similar trends were observed in global ASMR and ASDR, but the decline started in 2019 (Figures 5C, E).

As shown in the Figures 5B, D, F, in China, ASIR was relatively stable before 2005, then showed a rapid increase from 2005 to 2010 with an APC of 5.94 (95% CI: 5.66 ∼ 6.25), followed by a decline with an APC of −1.14 (95% CI: −1.26 ∼−1.03). The ASDR showed a declining trend that slowed down before 2006, rapidly increased to a high point between 2006 and 2009, and then rapidly declined after 2012. The ASMR trend was similar to ASDR, but it started to gradually increase around 1999. The specific numbers can be found in Supplementary Table 5.

### 3.4 Analysis of the burden of ILD in BRI countries and cross-border inequality

**FIGURE 6:**
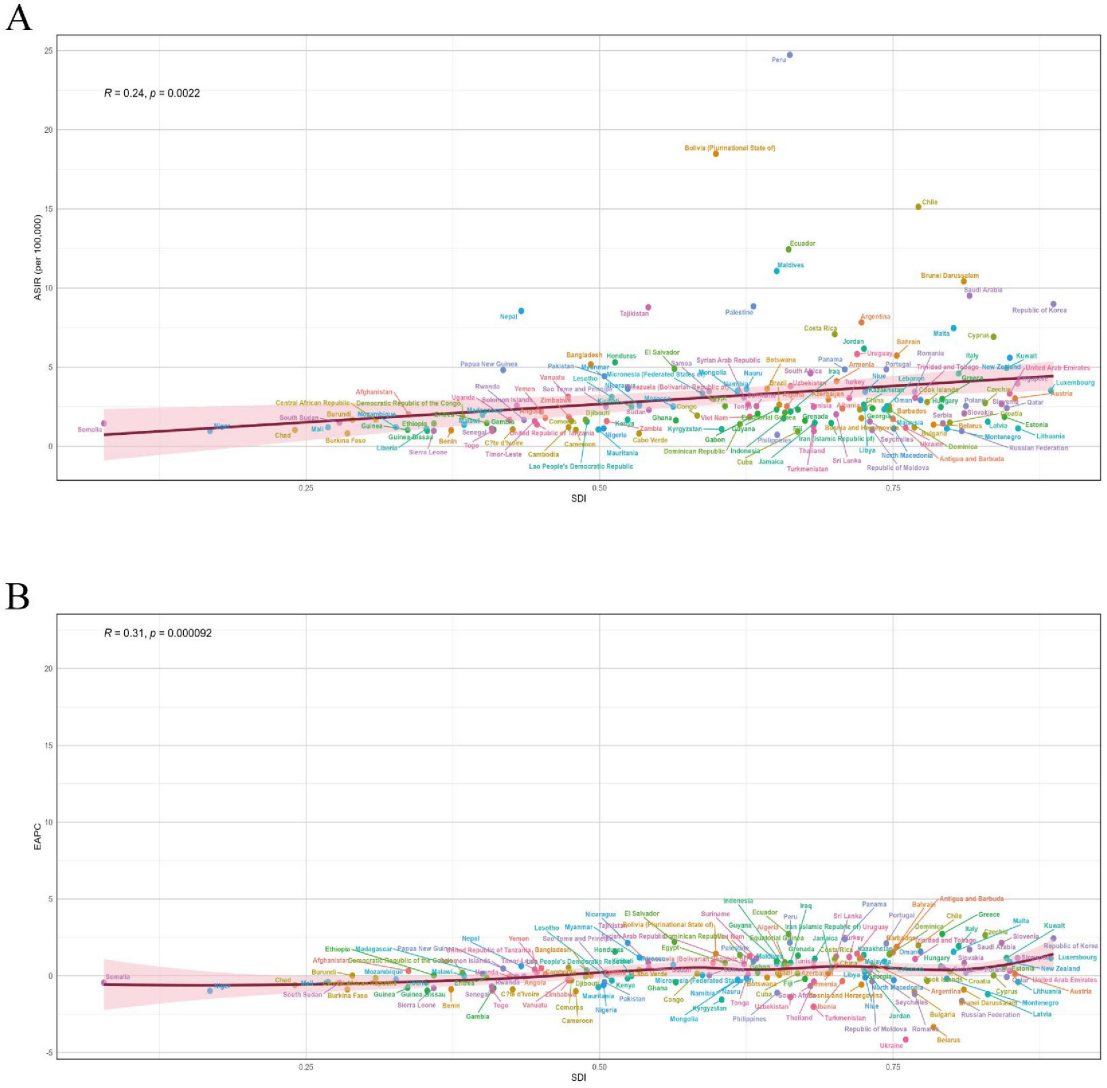
**(A)** Correlation between SDI and ILD ASIR in 2021; **(B)** correlation between SDI in 2021 and EAPC of ASIR from 1990 to 2021

A BRI country in 2021 had a positive correlation between SDI and ILD incidence rate (*R* = 0.24, *p* = 0.0022) (Figure 6A). This indicates that a higher level of socioeconomic development is associated with an increase in ILD incidence rate. Additionally, a positive correlation was observed between the EAPC of ILD incidence rate from 1990 to 2021 and SDI (*R* = 0.31, *p* < 0.001) (Figure 6B). This suggests that the annual average growth rate of ILD prevalence is faster in countries with a higher SDI.

**FIGURE 7:**
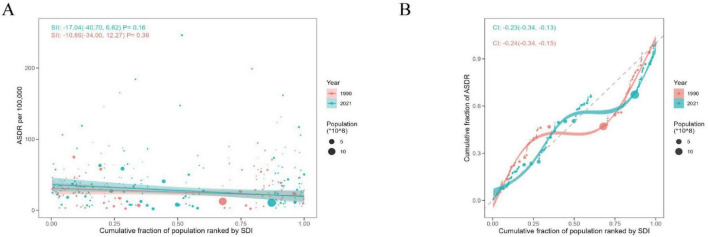
Health inequality regression curves and concentration curves for the ASDR of ILD in BRI countries, 1990 and 2021. **(A)** Slopes index of ILD; **(B)** health concentration index of ILD.

Observations (Figures 7A, B) show that from the slope index of inequality, the DALYs rate gap between countries with the highest and lowest SDI in 1990 and 2021 was −10.86 per 100,000 people (95% CI: −34.00, 12.27) and −17.04 per 100,000 (95% CI: −40.07, 6.26), respectively, indicating a negative correlation between ASDR and SDI, and that the inequality in ILD between high SDI countries and low SDI countries has been expanding over the past three decades, but to a small extent. In contrast, relative inequality analysis shows that the health concentration index remained almost unchanged between 1990 (−0.24, 95% CI: −0.34 to −0.15) and 2021 (−0.23, 95% CI: −0.34, −0.15).

### 3.5 Prediction of ILD burden by 2031

For global ASDR data, the auto.arima() function from the forecast package was used to screen and optimize the Male, Female, and Both models, with the optimized model choices being (2,2,0), (0,2,2), (2,2,0) (AIC = −127.66, −153.01, −146.05, BIC = −123.46, −148.8, −141.85, AICC = −126.74, −152.08, −145.13). The observed values and fitted values show good consistency (cor = 1, 1, 1, *P* = < 0001, <0001, <0001), and the residuals were found to be normally distributed based on Q-Q plots, ACF plots, and PACF plots (Supplementary Figure 1A). The Ljung-Box test confirms that the residuals of the models are white noise (*Q* = 2.6, 3.11, 2.11, *P* = 0.63, 0.54, 0.72). For Chinese ASDR data, the optimized model choices were (2,0,0), (1,1,0), (2,0,0) (AIC = −23.73, −71.13, −52.78, BIC = −17.87, −68.27, −46.91, AICC = −22.25, −70.7, −51.29). The observed values and fitted values show good consistency (cor = 0.98, 1, 0.99, *P* = < 0001, <0001, <0001), and the residuals were found to be normally distributed based on Q-Q plots, ACF plots, and PACF plots (Supplementary Figure 1B). The Ljung-Box test confirms that the residuals of the models are white noise (*Q* = 4.6, 4.6, 2.93, *P* = 0.33, 0.47, 0.57).

**FIGURE 8:**
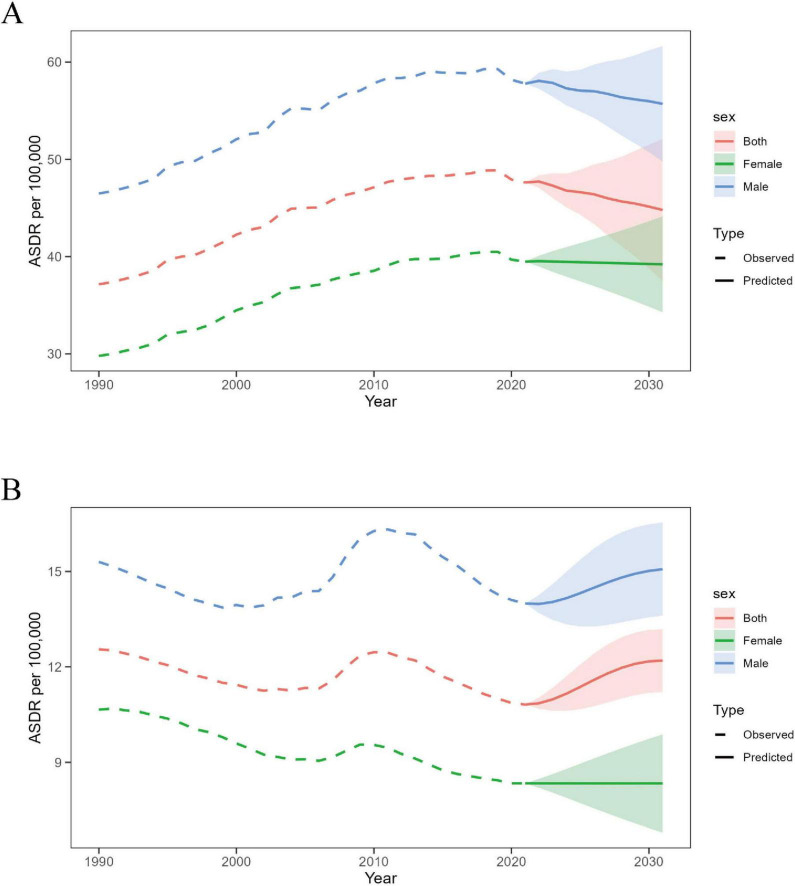
Prediction of ASMR trends for ILD by gender from 2022 to 2031. **(A)** Global; **(B)** China.

The prediction trend of ILD’s ASDR shows a decline from 2022 to 2031 globally. By 2031, the ASDR for males is expected to decrease to 55.7 per 100,000 people, for females to 39.2 per 100,000 people, and for both genders combined to 44.79 per 100,000 people. This means that compared to 47.62 per 100,000 people in 2021, the overall ASMR for all genders is expected to decrease by 5.94% (Figure 8A and Supplementary Table 6). In contrast, the prediction trend for future ASDR in Chinese males is expected to increase, reaching 15.07 per 100,000 people by 2031, while for females, it is expected to remain almost the same as in 2021, at 8.34 per 100,000 people (Figure 8B and Supplementary Table 6).

## 4 Discussion

In this study, we conducted a secondary analysis of the 2021 GBD data to provide a recent overview of the epidemiology of ILD globally, in China, and in BRI countries, and to make predictions for the next 11 years. It also reveals health inequalities and changes related to ILD over the past 30 years.

Chronic respiratory diseases are the third leading cause of death (20), but with continuous efforts in prevention, control, and treatment, all burden indicators for COPD, asthma, and pneumoconiosis have declined (21, 22). This study shows that from 1990 to 2021, the global and BRI countries’ ASIR, ASDR, and ASMR for ILD have generally increased. ILD, as a group of heterogeneous diseases, has very different prognoses, with some resolving without intervention and others progressing to the fibrotic stage, where current treatments cannot prevent the diseases from leading to respiratory failure and death (23). Among them, IPF has the worst prognosis, while Hypersensitivity Pneumonitis and Connective tissue disease-related ILD are relatively better (24). Given that the risk of ILD is very high in the elderly (5), with the continuous growth and aging of the global population, further growth seems inevitable. However, according to our global predictions, the burden of ILD is expected to reverse its previous trend. We speculate that this may be due to the widespread use of two antifibrotic therapies (pirfenidone and nintedanib), which have been proven to slow disease progression and improve survival rates (25–27). On the other hand, proactive diagnosis and prevention of ILD have played a significant role. The use of emerging technologies may also contribute to a reduction in the overall burden trend of ILD. The early radiological manifestation of ILD, interstitial lung abnormality (ILA), has recently gained increasing attention (28), although it was once difficult to recognize (29). With the intervention of AI tools, it is now possible to predict and assess the progression of ILD based on high-resolution computed tomography (HRCT) features that are difficult for the naked eye to detect (30).

Interstitial lung diseases burden heterogeneity is also noteworthy. In Andean Latin America, ASIR, ASDR, and ASMR are the highest among the 21 GBD regions, while the top three BRI countries with the highest ASIR, ASDR, and ASMR–Peru, Bolivia (Plurinational State of), and Chile–are Latin American nations. Smoking and environmental exposure are important risk factors for ILD (31, 32). Chile had the highest smoking rate in the region of the Americas in 2022, reaching 28.7% (33), which may be one reason for its high ILD burden. After implementing the Tobacco Law in 2013, the smoking rate in Chile has decreased, but it still faces challenges such as interference from the tobacco industry and difficulties in enforcement (34). Peru, Bolivia (Plurinational State of), and Chile all have abundant mineral resources. The public health situation in Peru and Bolivia (Plurinational State of) is concerning, with workplace exposure in mining leading to an increased likelihood of respiratory diseases such as IPF and pneumoconiosis (35, 36). Additionally, toxic metal exposure in mining areas causes health issues in surrounding regions (37). Therefore, ESG regulation of mining companies and promotion of dust-reducing mining technologies should be strengthened in these regions. Meanwhile, under the BRI framework, active international cooperation can be promoted to improve mineral extraction technology and enhance medical infrastructure.

On the other hand, we can observe that in global high SDI regions, the growth rates of ASIR, ASDR, and ASMR for ILD are the highest. Relatedness analysis also confirms that BRI countries are applicable to this conclusion. This phenomenon seems to be attributed to the relatively more abundant and accessible medical resources in high SDI regions, which facilitate the diagnosis of ILD and may lead to an increase in incidence (38), while over-medicalization and drug toxicity also contribute to the rise in incidence, such as the widespread use of immunosuppressants leading to an incidence rate of immunotherapy-related ILD of about 3.5% (39); on the other hand, high SDI regions also experience the aggravation of population aging, with a sharp increase in ILD incidence in the elderly population, who often have comorbidities such as cardiovascular disease and diabetes, which may accelerate the process of pulmonary fibrosis through chronic inflammation or metabolic abnormalities (40, 41), leading to an increase in ASDR and ASMR.

In terms of health inequalities among BRI countries, we calculated the inequality slope index and health concentration index based on the cross-national health gradient and SDI ranking. It can be observed that the health inequality of ILD is gradually widening among countries with different levels of economic development. Due to the treatment challenges of diseases like IPF, new treatment methods are continuously being developed (42, 43), but they are initially only used in more developed countries. Therefore, the health initiatives of BRI are more important, promoting the flow and sharing of medical resources, which is conducive to reducing health inequalities in ILD among countries. During the COVID-19 pandemic, China provided medical supplies to over 120 BRI countries, making up for the shortcomings of traditional global health governance.

China, as the initiator of the BRI, needs to be deeply understood in terms of changes in its ILD burden, which helps to understand its positioning in BRI countries and the role it should play. China’s ASIR for ILD is at a medium level among BRI countries, while ASDR and ASMR are smaller, ranking in the top 17%. In the Chinese population, ILD is mainly prevalent in the elderly, but the incidence in middle-aged groups is increasing. The concentration of DALYs appears 5–10 years earlier than deaths, so it is important to focus on the prevention and early diagnosis of ILD in middle-aged groups to address the issue of poor prognosis and reduce the burden of ILD. From a timeline perspective, ASIR began to increase after 2000, with a rapid increase around 2006. The main reason was the gradual deployment of high-resolution CT (HRCT) equipment in tertiary hospitals in China after 2000, which significantly improved the detection rate of ILD subtypes such as pulmonary fibrosis (44); the promotion of urban resident medical insurance in 2007 reduced the threshold for ILD patients to seek medical care, leading to more cases being included in statistics. At the same time, with the acceleration of industrialization, exposure to industrial dust (such as silica dust, asbestos), chemical smoke (such as VOCs), and PM2.5 pollutants increased (45), and studies show that traffic pollution (nitrogen oxides, carbon particles) and PM2.5 exposure are positively correlated with ILD risk (46, 47). Then, after around 2010, the incidence and mortality of ILD began to decrease, which may be related to China’s environmental governance. Studies have shown that the average PM2.5 pollution exposure level of the Chinese population decreased by 19.8 μg/m^3^ and 10.9 μg/m^3^ between 2013–2017 and 2018–2020, respectively (48); in 2011, the diagnostic process combining HRCT and pathological biopsy was introduced in guidelines (49), reducing misdiagnosis and overdiagnosis. We predict that in the next 10 years, as China’s aging population intensifies, its ASDR for ILD will slowly rise. To address this, the Chinese government has taken preemptive measures, issuing and implementing the Health China Action–Chronic Respiratory Disease Prevention and Control Action Implementation Plan (2024–2030) (50).

This study has some limitations: (1) Since ILD includes a wide range of diseases, its diagnostic and epidemiological characteristics vary significantly. As ILD subdivides, future studies may focus more on specific ILDs. (2) Currently, the GBD database lacks attribution data for ILD, such as smoking and environmental exposures, which makes it impossible to conduct further research on the causes of ILD. (3) Only a few countries or regions provide actual national data, while most countries lack reliable primary data sources, especially in low- and middle-income countries, so the burden estimates are heavily dependent on modeling data. Therefore, the interpretation of the burden at the national level has a certain degree of unreliability. More health surveys should be conducted at the national level to make the data more representative.

## 5 Summary

From 1990 to 2021, the global ASIR, ASMR, and ASDR of ILD have all increased. In 2021, High SDI regions had the highest ASIR, ASMR, and ASDR. Peru had the highest ASIR, ASMR, and ASDR among BRI countries. It is predicted that the global ASDR will show a slow downward trend in the future, while it may increase in China. Health inequality of ILD in BRI countries is gradually increasing, which may be caused by various factors such as population structure, environmental factors, and economic development. Currently, global isolationism is prevalent, and BRI can solve the anti-globalization dilemma through open cooperation. For example, promoting the construction of cross-border medical consortia, building a full range of ILD screening networks and big data centers in the BRI, and a special fund for the Healthy Silk Road to subsidize access to anti-fibrotic drugs in low-income BRI countries. BRI will contribute to a more inclusive and sustainable global development framework that can help reduce health inequalities among countries.

## Data Availability

Publicly available datasets were analyzed in this study. This data can be found here: https://ghdx.healthdata.org/gbd-results-tool.

## Footnotes

1
https://ghdx.healthdata.org/gbd-results-tool

2
https://surveillance.cancer.gov/joinpoint/
