# Supplementary material for: Epidemiological analysis and temporal trends of interstitial lung diseases in global, Chinese, and Belt and Road Initiative countries: 1990–2021
Source: Front Med (Lausanne). 2025 Aug 29;12:1620714. doi: 10.3389/fmed.2025.1620714 (PMC12425704; doi:10.3389/fmed.2025.1620714)
Supplement: Supplementary file 1 [file Table_1.docx]

**Supplementary materials:**

## Abbreviations:

ILD: Interstitial lung diseases

BRI: The Belt and Road Initiative

EAPC: Estimated annual percentage change

AAPC: Average annual percentage change

ASIR: Age-standardized incidence rate

ASMR: Age-standardized mortality rate

DALYs: Disability-adjusted life years

ASDR: Age-standardized DALYs rate

CI: Confidence interval

SDI: Socio-demographic index

UI: Uncertainty interval

Contents

[Abbreviations: 1](#_Toc205419459)

[Table S1 3](#_Toc205419460)

[Table S2 18](#_Toc205419461)

[Table S3 33](#_Toc205419462)

[Table S4 48](#_Toc205419463)

[Table S5 51](#_Toc205419464)

[Table S6. 53](#_Toc205419465)

[Figure S1 54](#_Toc205419466)

## Table S1 The incidence number, age-standardized rates and temporal trends of Interstitial lung diseases in the Belt and Road Initiative countries from 1990 to 2021.

| **location** |  | | | | |
| --- | --- | --- | --- | --- | --- |
|  | **Number of cases, 1990  (thousands)** | **Age-standardised rate per 100,000 population, 1990** | **Number of cases, 2021  (thousands)** | **Age-standardised rate per 100,000 population, 2021** | **Estimated annual percentage change, 1990-2021** |
| Global | 157.441 (136.251,179.472) | 3.767 (3.268,4.277) | 390.267 (346.393,433.403) | 4.545 (4.055,5.038) | 0.725 (0.626,0.824) |
| Female | 71.177 (61.733,81.297) | 3.226 (2.805,3.678) | 175.586 (155.725,195.607) | 3.886 (3.456,4.313) | 0.714 (0.628,0.801) |
| Male | 86.264 (74.477,98.468) | 4.481 (3.892,5.051) | 214.681 (190.533,238.498) | 5.365 (4.802,5.953) | 0.707 (0.596,0.818) |
| High SDI | 65.229 (56.925,73.998) | 6.202 (5.419,7.03) | 155.238 (137.458,174.122) | 8.187 (7.286,9.066) | 0.919 (0.793,1.046) |
| High-middle SDI | 25.616 (22.401,29.235) | 2.493 (2.195,2.833) | 56.001 (50.155,61.921) | 2.97 (2.684,3.266) | 0.845 (0.7,0.991) |
| Middle SDI | 30.615 (26.132,35.606) | 2.71 (2.35,3.096) | 89.561 (79.276,100.011) | 3.365 (2.998,3.731) | 0.913 (0.803,1.023) |
| Low-middle SDI | 28.179 (23.956,32.565) | 4.392 (3.756,5.001) | 70.99 (62.702,79.388) | 4.849 (4.276,5.42) | 0.378 (0.348,0.408) |
| Low SDI | 7.696 (6.516,8.958) | 3.185 (2.736,3.644) | 18.292 (16.089,20.678) | 3.325 (2.949,3.698) | 0.156 (0.127,0.185) |
| High-income North America | 28.333 (24.53,32.456) | 8.483 (7.369,9.706) | 66.609 (58.423,75.153) | 10.954 (9.727,12.202) | 0.749 (0.617,0.882) |
| Caribbean | 0.394 (0.344,0.448) | 1.416 (1.253,1.599) | 1.003 (0.917,1.094) | 1.893 (1.727,2.072) | 1.063 (0.979,1.147) |
| Andean Latin America | 2.44 (2.228,2.662) | 12.293 (11.132,13.456) | 11.838 (11.094,12.531) | 20.467 (19.15,21.686) | 2.126 (1.972,2.281) |
| Central Latin America | 3.384 (2.932,3.876) | 3.607 (3.143,4.103) | 12.172 (10.943,13.423) | 4.809 (4.325,5.293) | 0.947 (0.86,1.034) |
| Tropical Latin America | 2.58 (2.184,3.04) | 2.436 (2.083,2.82) | 6.64 (5.821,7.441) | 2.617 (2.291,2.941) | 0.089 (0.018,0.161) |
| North Africa and Middle East | 4.326 (3.72,5.016) | 2.189 (1.906,2.488) | 15.113 (13.574,16.831) | 2.853 (2.578,3.151) | 0.98 (0.924,1.037) |
| South Asia | 35.968 (30.515,41.709) | 6.005 (5.109,6.881) | 96.281 (84.137,108.635) | 6.439 (5.65,7.262) | 0.249 (0.221,0.276) |
| Central Sub-Saharan Africa | 0.456 (0.385,0.531) | 1.797 (1.547,2.053) | 1.272 (1.103,1.455) | 1.879 (1.662,2.097) | 0.166 (0.13,0.203) |
| Eastern Sub-Saharan Africa | 1.328 (1.107,1.559) | 1.522 (1.306,1.739) | 3.244 (2.78,3.753) | 1.528 (1.344,1.724) | -0.018 (-0.031,-0.006) |
| Southern Sub-Saharan Africa | 1.381 (1.182,1.597) | 4.678 (4.002,5.361) | 2.577 (2.241,2.928) | 4.21 (3.686,4.73) | -0.518 (-0.683,-0.353) |
| Western Sub-Saharan Africa | 1.436 (1.204,1.682) | 1.369 (1.172,1.576) | 2.981 (2.544,3.486) | 1.13 (0.992,1.28) | -0.649 (-0.718,-0.58) |
| Oceania | 0.155 (0.139,0.174) | 3.717 (3.373,4.087) | 0.419 (0.385,0.456) | 4.2 (3.906,4.509) | 0.385 (0.36,0.41) |
| Central Asia | 1.672 (1.504,1.851) | 3.358 (3.055,3.698) | 2.849 (2.622,3.094) | 3.38 (3.124,3.647) | 0.016 (-0.212,0.245) |
| Central Europe | 3.65 (3.251,4.1) | 2.585 (2.299,2.905) | 4.034 (3.661,4.443) | 2.425 (2.201,2.687) | 0.016 (-0.072,0.105) |
| East Asia | 19.463 (16.281,23.141) | 1.898 (1.605,2.227) | 50.031 (42.927,57.6) | 2.313 (2.022,2.639) | 1.122 (0.854,1.391) |
| Eastern Europe | 5.322 (4.51,6.209) | 2.07 (1.781,2.401) | 2.682 (2.299,3.096) | 1.04 (0.889,1.211) | -2.563 (-2.681,-2.445) |
| High-income Asia Pacific | 18.818 (16.024,22.064) | 9.151 (7.819,10.656) | 43.787 (38.399,49.65) | 11.595 (10.25,13.023) | 0.787 (0.595,0.978) |
| Australasia | 0.798 (0.717,0.885) | 3.429 (3.078,3.798) | 3.392 (3.07,3.718) | 6.446 (5.879,7.02) | 2.15 (1.926,2.373) |
| Western Europe | 18.948 (17.007,20.974) | 3.644 (3.264,4.051) | 43.62 (39.614,47.749) | 5.298 (4.82,5.807) | 1.456 (1.282,1.63) |
| Southeast Asia | 3.839 (3.218,4.512) | 1.36 (1.164,1.564) | 11.245 (9.857,12.738) | 1.617 (1.424,1.809) | 0.545 (0.531,0.559) |
| Southern Latin America | 2.75 (2.529,2.975) | 5.988 (5.513,6.474) | 8.477 (7.913,9.062) | 9.904 (9.26,10.572) | 1.676 (1.527,1.826) |
| China | 18.993 (15.851,22.605) | 1.919 (1.619,2.255) | 48.514 (41.541,55.949) | 2.323 (2.027,2.653) | 1.106 (0.832,1.381) |
| Armenia | 0.11 (0.098,0.121) | 3.84 (3.482,4.21) | 0.171 (0.157,0.187) | 4.112 (3.765,4.481) | 0.671 (0.439,0.905) |
| Azerbaijan | 0.157 (0.139,0.175) | 2.913 (2.613,3.239) | 0.302 (0.271,0.335) | 2.962 (2.676,3.273) | 0.164 (-0.07,0.399) |
| Georgia | 0.137 (0.123,0.151) | 2.264 (2.047,2.516) | 0.131 (0.122,0.141) | 2.407 (2.231,2.592) | 0.625 (0.416,0.834) |
| Kazakhstan | 0.263 (0.231,0.298) | 1.907 (1.695,2.144) | 0.498 (0.454,0.546) | 2.632 (2.409,2.876) | 1.352 (0.937,1.768) |
| Kyrgyzstan | 0.051 (0.044,0.059) | 1.569 (1.371,1.78) | 0.064 (0.053,0.075) | 1.08 (0.929,1.242) | -1.549 (-1.645,-1.453) |
| Mongolia | 0.039 (0.034,0.043) | 3.454 (3.1,3.817) | 0.079 (0.072,0.088) | 3.319 (3.029,3.647) | -0.266 (-0.386,-0.146) |
| Tajikistan | 0.208 (0.187,0.229) | 7.293 (6.523,8.035) | 0.509 (0.467,0.552) | 8.783 (8.017,9.547) | 0.848 (0.6,1.096) |
| Turkmenistan | 0.051 (0.045,0.058) | 2.353 (2.104,2.613) | 0.058 (0.05,0.067) | 1.269 (1.111,1.447) | -2.014 (-2.272,-1.757) |
| Uzbekistan | 0.657 (0.595,0.726) | 5.231 (4.732,5.724) | 1.036 (0.955,1.13) | 3.801 (3.526,4.076) | -1.386 (-1.635,-1.137) |
| Brunei Darussalam | 0.016 (0.014,0.018) | 12.981 (11.699,14.393) | 0.042 (0.039,0.046) | 10.423 (9.65,11.335) | -0.902 (-0.988,-0.816) |
| Republic of Korea | 1.904 (1.696,2.16) | 5.031 (4.552,5.595) | 7.893 (7.171,8.673) | 8.991 (8.212,9.832) | 2.438 (2.173,2.704) |
| Singapore | 0.086 (0.075,0.099) | 2.969 (2.637,3.362) | 0.331 (0.301,0.363) | 3.964 (3.622,4.322) | 1.163 (1.045,1.281) |
| Afghanistan | 0.126 (0.107,0.147) | 1.887 (1.612,2.179) | 0.294 (0.25,0.346) | 2.021 (1.78,2.26) | 0.318 (0.247,0.389) |
| Bahrain | 0.008 (0.007,0.01) | 3.559 (3.189,3.948) | 0.06 (0.054,0.067) | 5.731 (5.241,6.261) | 1.889 (1.718,2.06) |
| Iran (Islamic Republic of) | 0.421 (0.341,0.506) | 1.269 (1.052,1.499) | 1.282 (1.093,1.5) | 1.475 (1.267,1.688) | 0.531 (0.463,0.6) |
| Iraq | 0.177 (0.149,0.209) | 1.765 (1.52,2.025) | 0.662 (0.586,0.753) | 2.195 (1.972,2.441) | 0.848 (0.783,0.913) |
| Jordan | 0.087 (0.079,0.096) | 5.37 (4.865,5.904) | 0.541 (0.496,0.584) | 6.166 (5.717,6.621) | 0.472 (0.45,0.494) |
| Kuwait | 0.032 (0.028,0.038) | 3.702 (3.367,4.041) | 0.183 (0.164,0.204) | 4.945 (4.618,5.294) | 1.156 (0.997,1.314) |
| Lebanon | 0.05 (0.043,0.059) | 2.17 (1.862,2.499) | 0.152 (0.135,0.171) | 2.525 (2.239,2.851) | 0.569 (0.507,0.63) |
| Oman | 0.02 (0.017,0.024) | 1.936 (1.681,2.217) | 0.095 (0.082,0.112) | 2.922 (2.617,3.264) | 1.577 (1.422,1.732) |
| Palestine | 0.066 (0.06,0.073) | 6.908 (6.246,7.614) | 0.276 (0.255,0.297) | 8.845 (8.134,9.49) | 0.94 (0.79,1.091) |
| Qatar | 0.007 (0.006,0.008) | 2.437 (2.151,2.743) | 0.058 (0.048,0.07) | 2.42 (2.19,2.675) | -0.076 (-0.244,0.091) |
| Saudi Arabia | 0.405 (0.363,0.449) | 5.954 (5.329,6.578) | 2.494 (2.266,2.737) | 9.516 (8.734,10.313) | 1.708 (1.612,1.805) |
| Syrian Arab Republic | 0.153 (0.131,0.177) | 2.462 (2.141,2.791) | 0.422 (0.376,0.475) | 3.092 (2.791,3.423) | 0.764 (0.703,0.825) |
| Turkey | 0.936 (0.819,1.066) | 2.326 (2.054,2.616) | 2.909 (2.642,3.19) | 3.054 (2.793,3.356) | 1.223 (1.05,1.397) |
| United Arab Emirates | 0.031 (0.026,0.038) | 2.975 (2.558,3.394) | 0.351 (0.291,0.416) | 3.331 (2.951,3.684) | 0.333 (0.271,0.395) |
| Yemen | 0.119 (0.099,0.141) | 1.971 (1.667,2.267) | 0.407 (0.349,0.472) | 2.198 (1.938,2.447) | 0.493 (0.399,0.588) |
| Bangladesh | 2.495 (2.224,2.775) | 4.906 (4.373,5.447) | 7.446 (6.712,8.19) | 5.173 (4.672,5.66) | 0.027 (-0.018,0.071) |
| Nepal | 0.631 (0.559,0.713) | 6.559 (5.841,7.37) | 2.035 (1.852,2.214) | 8.553 (7.79,9.282) | 0.635 (0.383,0.887) |
| Pakistan | 2.887 (2.44,3.341) | 4.935 (4.186,5.706) | 5.643 (4.934,6.381) | 4.428 (3.87,5.021) | -0.413 (-0.482,-0.344) |
| Cambodia | 0.052 (0.043,0.062) | 1.044 (0.889,1.199) | 0.161 (0.138,0.186) | 1.211 (1.059,1.371) | 0.532 (0.503,0.56) |
| Indonesia | 1.607 (1.324,1.918) | 1.448 (1.22,1.691) | 4.551 (3.927,5.252) | 1.771 (1.533,2.011) | 0.63 (0.562,0.698) |
| Lao People's Democratic Republic | 0.03 (0.025,0.035) | 1.387 (1.193,1.574) | 0.08 (0.07,0.091) | 1.557 (1.374,1.734) | 0.343 (0.309,0.377) |
| Malaysia | 0.187 (0.164,0.212) | 1.804 (1.615,2.027) | 0.699 (0.633,0.773) | 2.336 (2.118,2.572) | 0.927 (0.901,0.953) |
| Maldives | 0.009 (0.008,0.01) | 8.537 (7.687,9.401) | 0.046 (0.042,0.05) | 11.072 (10.199,11.976) | 0.789 (0.758,0.821) |
| Myanmar | 0.432 (0.37,0.498) | 1.806 (1.559,2.048) | 1.315 (1.167,1.482) | 2.572 (2.29,2.88) | 1.201 (1.071,1.331) |
| Philippines | 0.336 (0.272,0.408) | 0.964 (0.8,1.133) | 0.676 (0.556,0.81) | 0.728 (0.614,0.852) | -1.103 (-1.173,-1.033) |
| Sri Lanka | 0.167 (0.145,0.191) | 1.412 (1.252,1.588) | 0.534 (0.478,0.591) | 2.02 (1.822,2.23) | 1.258 (1.23,1.285) |
| Thailand | 0.424 (0.356,0.502) | 1.016 (0.876,1.17) | 0.992 (0.863,1.127) | 0.963 (0.842,1.103) | -0.369 (-0.453,-0.285) |
| Timor-Leste | 0.005 (0.004,0.006) | 1.433 (1.236,1.641) | 0.014 (0.012,0.016) | 1.598 (1.421,1.787) | 0.408 (0.36,0.456) |
| Viet Nam | 0.544 (0.457,0.638) | 1.301 (1.109,1.494) | 1.958 (1.707,2.212) | 1.863 (1.645,2.085) | 1.281 (1.207,1.354) |
| Albania | 0.068 (0.06,0.078) | 2.718 (2.413,3.03) | 0.089 (0.08,0.098) | 2.386 (2.144,2.66) | -0.346 (-0.46,-0.232) |
| Bosnia and Herzegovina | 0.096 (0.083,0.111) | 2.132 (1.847,2.442) | 0.085 (0.076,0.093) | 1.777 (1.586,2.002) | -0.573 (-0.714,-0.431) |
| Bulgaria | 0.166 (0.149,0.186) | 1.598 (1.415,1.823) | 0.109 (0.098,0.122) | 1.192 (1.042,1.373) | -1.058 (-1.089,-1.028) |
| Croatia | 0.077 (0.065,0.089) | 1.369 (1.167,1.602) | 0.079 (0.071,0.087) | 1.497 (1.331,1.686) | 0.289 (-0.201,0.782) |
| Czechia | 0.174 (0.152,0.197) | 1.451 (1.263,1.671) | 0.447 (0.408,0.492) | 2.736 (2.497,3.015) | 2.637 (2.34,2.935) |
| Hungary | 0.287 (0.262,0.316) | 2.254 (2.046,2.495) | 0.36 (0.334,0.386) | 2.488 (2.29,2.711) | 0.602 (0.499,0.706) |
| North Macedonia | 0.025 (0.021,0.03) | 1.241 (1.058,1.449) | 0.031 (0.027,0.035) | 1.127 (0.981,1.305) | -0.281 (-0.374,-0.187) |
| Montenegro | 0.008 (0.007,0.009) | 1.224 (1.035,1.432) | 0.008 (0.007,0.01) | 1.117 (0.966,1.297) | -0.195 (-0.281,-0.109) |
| Poland | 1.067 (0.897,1.249) | 2.569 (2.177,3.013) | 1.337 (1.182,1.52) | 2.545 (2.26,2.892) | 0.212 (0.113,0.31) |
| Romania | 1.337 (1.209,1.483) | 5.056 (4.594,5.529) | 1.005 (0.916,1.095) | 3.443 (3.146,3.744) | -1.188 (-1.364,-1.012) |
| Serbia | 0.151 (0.132,0.173) | 1.371 (1.198,1.581) | 0.18 (0.164,0.198) | 1.452 (1.303,1.632) | 0.545 (0.317,0.772) |
| Slovakia | 0.099 (0.085,0.113) | 1.742 (1.509,2.002) | 0.156 (0.142,0.17) | 2.077 (1.884,2.311) | 0.824 (0.731,0.917) |
| Slovenia | 0.037 (0.033,0.042) | 1.641 (1.444,1.879) | 0.09 (0.081,0.099) | 2.666 (2.399,2.966) | 2.148 (1.683,2.615) |
| Belarus | 0.411 (0.37,0.457) | 3.435 (3.103,3.801) | 0.156 (0.138,0.174) | 1.369 (1.212,1.544) | -3.342 (-3.481,-3.203) |
| Estonia | 0.028 (0.025,0.032) | 1.631 (1.43,1.851) | 0.031 (0.029,0.034) | 1.862 (1.692,2.056) | 0.57 (0.517,0.624) |
| Latvia | 0.077 (0.07,0.086) | 2.534 (2.257,2.821) | 0.037 (0.033,0.04) | 1.556 (1.378,1.751) | -1.196 (-1.453,-0.938) |
| Lithuania | 0.052 (0.045,0.059) | 1.274 (1.104,1.474) | 0.039 (0.035,0.043) | 1.135 (0.999,1.286) | -0.399 (-0.688,-0.11) |
| Republic of Moldova | 0.053 (0.044,0.063) | 1.168 (0.986,1.379) | 0.045 (0.039,0.052) | 1.024 (0.88,1.192) | -0.368 (-0.458,-0.277) |
| Russian Federation | 2.485 (2.057,2.917) | 1.477 (1.235,1.739) | 1.707 (1.446,1.985) | 0.961 (0.809,1.127) | -1.641 (-1.716,-1.565) |
| Ukraine | 2.216 (1.88,2.58) | 3.502 (3.01,4.072) | 0.668 (0.574,0.77) | 1.173 (1.007,1.361) | -4.146 (-4.379,-3.913) |
| Austria | 0.287 (0.262,0.319) | 2.904 (2.635,3.248) | 0.434 (0.402,0.468) | 3.025 (2.784,3.287) | 0.118 (-0.158,0.395) |
| Cyprus | 0.056 (0.049,0.063) | 6.715 (5.958,7.516) | 0.143 (0.128,0.158) | 6.92 (6.281,7.588) | 0.029 (-0.163,0.221) |
| Greece | 0.218 (0.192,0.246) | 1.559 (1.377,1.764) | 0.609 (0.548,0.669) | 2.993 (2.715,3.285) | 2.722 (2.514,2.93) |
| Italy | 2.172 (1.836,2.538) | 2.895 (2.434,3.416) | 5.326 (4.613,6.036) | 4.599 (4.052,5.191) | 1.934 (1.368,2.503) |
| Luxembourg | 0.013 (0.012,0.014) | 2.555 (2.319,2.817) | 0.035 (0.032,0.038) | 3.521 (3.25,3.815) | 1.137 (0.864,1.41) |
| Malta | 0.02 (0.019,0.022) | 4.796 (4.42,5.196) | 0.066 (0.06,0.071) | 7.464 (6.944,7.989) | 1.56 (1.362,1.759) |
| Portugal | 0.349 (0.308,0.395) | 2.662 (2.357,2.981) | 1.048 (0.962,1.129) | 4.863 (4.499,5.238) | 2.106 (1.89,2.323) |
| Algeria | 0.306 (0.259,0.36) | 2.177 (1.867,2.487) | 0.992 (0.878,1.134) | 2.524 (2.248,2.827) | 0.585 (0.538,0.632) |
| Egypt | 0.653 (0.543,0.774) | 2.012 (1.715,2.303) | 1.931 (1.69,2.222) | 2.54 (2.252,2.877) | 0.833 (0.789,0.876) |
| Libya | 0.052 (0.045,0.061) | 2.279 (1.948,2.626) | 0.154 (0.135,0.176) | 2.393 (2.125,2.687) | 0.253 (0.162,0.344) |
| Morocco | 0.335 (0.283,0.392) | 2.059 (1.765,2.368) | 0.911 (0.81,1.027) | 2.515 (2.235,2.811) | 0.716 (0.676,0.756) |
| Tunisia | 0.118 (0.1,0.138) | 2.123 (1.821,2.448) | 0.335 (0.295,0.377) | 2.501 (2.208,2.804) | 0.591 (0.551,0.63) |
| Angola | 0.079 (0.067,0.092) | 1.73 (1.489,1.974) | 0.268 (0.234,0.308) | 1.801 (1.614,2.004) | 0.12 (0.091,0.149) |
| Central African Republic | 0.023 (0.019,0.027) | 1.781 (1.524,2.042) | 0.045 (0.039,0.053) | 1.693 (1.485,1.901) | -0.167 (-0.199,-0.134) |
| Congo | 0.022 (0.018,0.025) | 1.844 (1.595,2.09) | 0.064 (0.056,0.073) | 1.944 (1.745,2.152) | 0.154 (0.115,0.193) |
| Democratic Republic of the Congo | 0.318 (0.267,0.369) | 1.81 (1.555,2.07) | 0.855 (0.736,0.984) | 1.901 (1.666,2.133) | 0.194 (0.149,0.239) |
| Equatorial Guinea | 0.004 (0.003,0.004) | 1.748 (1.504,2.013) | 0.015 (0.013,0.017) | 2.127 (1.889,2.366) | 0.87 (0.784,0.956) |
| Gabon | 0.011 (0.01,0.013) | 1.908 (1.673,2.166) | 0.025 (0.022,0.028) | 2.06 (1.853,2.273) | 0.212 (0.188,0.236) |
| Burundi | 0.044 (0.037,0.052) | 1.665 (1.431,1.898) | 0.102 (0.087,0.118) | 1.673 (1.479,1.878) | 0.01 (-0.007,0.027) |
| Comoros | 0.004 (0.003,0.004) | 1.721 (1.489,1.959) | 0.009 (0.008,0.01) | 1.598 (1.419,1.787) | -0.292 (-0.319,-0.266) |
| Djibouti | 0.003 (0.003,0.004) | 1.662 (1.439,1.891) | 0.013 (0.012,0.016) | 1.673 (1.501,1.859) | -0.036 (-0.081,0.009) |
| Eritrea | 0.022 (0.018,0.026) | 1.488 (1.292,1.695) | 0.054 (0.047,0.062) | 1.474 (1.318,1.646) | -0.128 (-0.163,-0.094) |
| Ethiopia | 0.33 (0.269,0.392) | 1.435 (1.211,1.664) | 0.769 (0.654,0.891) | 1.445 (1.254,1.635) | 0.032 (-0.016,0.079) |
| Kenya | 0.168 (0.14,0.198) | 1.671 (1.413,1.93) | 0.476 (0.405,0.556) | 1.686 (1.464,1.917) | -0.167 (-0.238,-0.095) |
| Madagascar | 0.105 (0.09,0.122) | 1.855 (1.616,2.101) | 0.273 (0.237,0.311) | 1.99 (1.772,2.219) | 0.133 (0.094,0.172) |
| Malawi | 0.063 (0.052,0.074) | 1.363 (1.17,1.554) | 0.132 (0.113,0.154) | 1.362 (1.207,1.532) | -0.013 (-0.025,0) |
| Mozambique | 0.087 (0.072,0.103) | 1.26 (1.073,1.452) | 0.177 (0.149,0.208) | 1.202 (1.052,1.366) | -0.241 (-0.31,-0.171) |
| Rwanda | 0.054 (0.046,0.064) | 1.669 (1.441,1.91) | 0.13 (0.112,0.149) | 1.671 (1.475,1.88) | -0.04 (-0.121,0.041) |
| Seychelles | 0.001 (0.001,0.001) | 1.348 (1.153,1.549) | 0.002 (0.002,0.002) | 1.566 (1.389,1.755) | 0.362 (0.287,0.438) |
| Somalia | 0.051 (0.042,0.061) | 1.657 (1.416,1.899) | 0.123 (0.103,0.145) | 1.45 (1.272,1.638) | -0.448 (-0.467,-0.428) |
| United Republic of Tanzania | 0.166 (0.139,0.194) | 1.303 (1.118,1.484) | 0.443 (0.382,0.513) | 1.375 (1.214,1.537) | 0.205 (0.167,0.243) |
| Uganda | 0.127 (0.108,0.149) | 1.706 (1.471,1.947) | 0.322 (0.278,0.37) | 1.667 (1.482,1.861) | -0.073 (-0.15,0.004) |
| Zambia | 0.052 (0.044,0.061) | 1.537 (1.336,1.748) | 0.148 (0.128,0.171) | 1.588 (1.411,1.77) | 0.074 (0.022,0.125) |
| Botswana | 0.023 (0.02,0.026) | 3.674 (3.218,4.098) | 0.063 (0.056,0.07) | 3.634 (3.308,3.962) | -0.109 (-0.186,-0.031) |
| Lesotho | 0.03 (0.026,0.035) | 3.389 (2.954,3.864) | 0.037 (0.033,0.042) | 3.11 (2.822,3.426) | -0.314 (-0.352,-0.276) |
| Namibia | 0.026 (0.023,0.03) | 3.714 (3.269,4.165) | 0.055 (0.05,0.061) | 3.514 (3.206,3.83) | -0.263 (-0.314,-0.213) |
| South Africa | 1.184 (1.012,1.372) | 5.291 (4.496,6.09) | 2.234 (1.937,2.541) | 4.611 (4.009,5.211) | -0.66 (-0.842,-0.478) |
| Zimbabwe | 0.105 (0.088,0.124) | 2.023 (1.757,2.299) | 0.167 (0.143,0.196) | 1.866 (1.648,2.087) | -0.3 (-0.353,-0.247) |
| Benin | 0.031 (0.027,0.036) | 1.322 (1.154,1.506) | 0.073 (0.062,0.086) | 1.012 (0.894,1.137) | -0.877 (-0.967,-0.786) |
| Burkina Faso | 0.053 (0.044,0.062) | 1.046 (0.893,1.211) | 0.104 (0.086,0.124) | 0.813 (0.7,0.932) | -0.884 (-0.927,-0.841) |
| Cameroon | 0.076 (0.065,0.087) | 1.402 (1.228,1.596) | 0.186 (0.158,0.218) | 1.039 (0.92,1.17) | -1.003 (-1.128,-0.879) |
| Cabo Verde | 0.002 (0.001,0.002) | 0.733 (0.607,0.869) | 0.004 (0.004,0.005) | 0.808 (0.697,0.93) | 0.391 (0.339,0.443) |
| Chad | 0.04 (0.034,0.046) | 1.244 (1.07,1.413) | 0.083 (0.07,0.098) | 1.029 (0.908,1.171) | -0.596 (-0.67,-0.522) |
| Côte d'Ivoire | 0.077 (0.064,0.092) | 1.39 (1.21,1.591) | 0.173 (0.147,0.202) | 1.071 (0.949,1.2) | -0.881 (-1.026,-0.737) |
| Gambia | 0.006 (0.005,0.007) | 1.319 (1.145,1.505) | 0.014 (0.012,0.017) | 1.053 (0.932,1.191) | -0.802 (-0.904,-0.701) |
| Ghana | 0.133 (0.115,0.152) | 1.776 (1.556,2.017) | 0.337 (0.297,0.38) | 1.64 (1.489,1.806) | -0.421 (-0.631,-0.211) |
| Guinea | 0.049 (0.042,0.056) | 1.335 (1.164,1.518) | 0.077 (0.065,0.09) | 1.045 (0.927,1.177) | -0.775 (-0.889,-0.662) |
| Guinea-Bissau | 0.007 (0.006,0.008) | 1.331 (1.156,1.524) | 0.011 (0.009,0.013) | 0.99 (0.873,1.121) | -0.966 (-1.081,-0.852) |
| Liberia | 0.017 (0.014,0.02) | 1.298 (1.105,1.493) | 0.033 (0.027,0.039) | 1.116 (0.97,1.275) | -0.446 (-0.508,-0.384) |
| Mali | 0.066 (0.056,0.077) | 1.357 (1.167,1.557) | 0.149 (0.129,0.17) | 1.208 (1.083,1.347) | -0.403 (-0.469,-0.337) |
| Mauritania | 0.015 (0.013,0.018) | 1.323 (1.149,1.513) | 0.028 (0.025,0.033) | 1.063 (0.946,1.186) | -0.738 (-0.855,-0.62) |
| Niger | 0.048 (0.04,0.056) | 1.318 (1.143,1.509) | 0.11 (0.093,0.131) | 0.974 (0.854,1.104) | -0.978 (-1.061,-0.894) |
| Nigeria | 0.712 (0.584,0.844) | 1.366 (1.144,1.603) | 1.382 (1.16,1.645) | 1.132 (0.973,1.298) | -0.603 (-0.63,-0.576) |
| Sao Tome and Principe | 0.002 (0.001,0.002) | 2.32 (2.05,2.615) | 0.004 (0.003,0.004) | 2.505 (2.29,2.758) | 0.165 (0.031,0.298) |
| Senegal | 0.053 (0.045,0.061) | 1.388 (1.213,1.576) | 0.109 (0.094,0.126) | 1.121 (0.989,1.25) | -0.683 (-0.797,-0.568) |
| Sierra Leone | 0.03 (0.025,0.035) | 1.26 (1.085,1.438) | 0.051 (0.043,0.06) | 0.982 (0.862,1.115) | -0.795 (-0.929,-0.66) |
| Togo | 0.021 (0.018,0.025) | 1.281 (1.115,1.464) | 0.052 (0.044,0.061) | 0.972 (0.855,1.109) | -0.949 (-1.073,-0.825) |
| South Sudan | 0.05 (0.043,0.059) | 1.735 (1.511,1.981) | 0.069 (0.059,0.079) | 1.499 (1.329,1.666) | -0.462 (-0.482,-0.442) |
| Sudan | 0.219 (0.183,0.259) | 2.021 (1.725,2.353) | 0.592 (0.515,0.68) | 2.304 (2.053,2.574) | 0.502 (0.445,0.56) |
| Guyana | 0.008 (0.007,0.009) | 1.806 (1.627,1.993) | 0.016 (0.014,0.017) | 2.326 (2.152,2.501) | 0.946 (0.892,1) |
| Suriname | 0.005 (0.005,0.006) | 1.893 (1.689,2.11) | 0.016 (0.015,0.017) | 2.539 (2.334,2.751) | 1.131 (1.061,1.202) |
| Bolivia (Plurinational State of) | 0.369 (0.332,0.407) | 12.585 (11.259,13.894) | 1.571 (1.442,1.706) | 18.483 (16.968,20.019) | 1.433 (1.378,1.488) |
| Ecuador | 0.348 (0.315,0.383) | 6.78 (6.109,7.485) | 1.969 (1.824,2.122) | 12.44 (11.515,13.406) | 2.725 (2.479,2.972) |
| Peru | 1.723 (1.574,1.879) | 14.632 (13.287,15.979) | 8.297 (7.815,8.783) | 24.725 (23.235,26.217) | 2.163 (2.007,2.319) |
| Venezuela (Bolivarian Republic of) | 0.266 (0.236,0.299) | 2.399 (2.165,2.641) | 0.905 (0.839,0.975) | 3 (2.782,3.226) | 0.839 (0.747,0.931) |
| Brazil | 2.545 (2.152,2.999) | 2.461 (2.103,2.85) | 6.513 (5.705,7.309) | 2.627 (2.298,2.955) | 0.063 (-0.011,0.138) |
| Argentina | 1.723 (1.567,1.881) | 5.42 (4.942,5.911) | 4.305 (4.026,4.571) | 7.832 (7.347,8.323) | 1.15 (0.964,1.337) |
| Chile | 0.884 (0.826,0.947) | 8.64 (8.079,9.22) | 3.865 (3.583,4.144) | 15.128 (14.031,16.231) | 1.98 (1.834,2.127) |
| Uruguay | 0.142 (0.126,0.159) | 3.868 (3.426,4.323) | 0.307 (0.286,0.329) | 5.83 (5.441,6.257) | 1.443 (1.343,1.543) |
| Fiji | 0.01 (0.008,0.012) | 1.725 (1.521,1.972) | 0.014 (0.013,0.016) | 1.617 (1.448,1.816) | -0.203 (-0.238,-0.169) |
| Kiribati | 0.001 (0.001,0.002) | 2.534 (2.304,2.791) | 0.002 (0.002,0.003) | 2.505 (2.312,2.731) | -0.004 (-0.035,0.028) |
| Micronesia (Federated States of) | 0.002 (0.002,0.003) | 3.285 (2.999,3.628) | 0.003 (0.003,0.003) | 3.352 (3.086,3.636) | 0.048 (0.008,0.088) |
| Papua New Guinea | 0.104 (0.093,0.118) | 4.188 (3.806,4.615) | 0.334 (0.307,0.363) | 4.822 (4.48,5.186) | 0.472 (0.452,0.493) |
| Samoa | 0.004 (0.004,0.004) | 3.435 (3.136,3.766) | 0.006 (0.006,0.007) | 3.482 (3.215,3.756) | 0.034 (-0.006,0.073) |
| Solomon Islands | 0.005 (0.004,0.006) | 2.584 (2.308,2.886) | 0.013 (0.011,0.014) | 2.582 (2.366,2.818) | -0.079 (-0.105,-0.053) |
| Tonga | 0.002 (0.002,0.002) | 2.555 (2.298,2.844) | 0.002 (0.002,0.003) | 2.661 (2.448,2.895) | 0.112 (0.088,0.135) |
| Vanuatu | 0.003 (0.003,0.003) | 3.277 (2.966,3.642) | 0.008 (0.007,0.008) | 3.133 (2.898,3.381) | -0.224 (-0.249,-0.199) |
| Cook Islands | 0 (0,0.001) | 3.033 (2.756,3.355) | 0.001 (0.001,0.001) | 2.798 (2.541,3.075) | -0.334 (-0.362,-0.307) |
| Nauru | 0 (0,0) | 3.783 (3.445,4.166) | 0 (0,0) | 3.61 (3.329,3.899) | -0.148 (-0.238,-0.059) |
| Niue | 0 (0,0) | 3.536 (3.226,3.885) | 0 (0,0) | 3.452 (3.159,3.76) | -0.102 (-0.127,-0.077) |
| New Zealand | 0.166 (0.14,0.195) | 4.291 (3.64,5.004) | 0.467 (0.404,0.532) | 5.592 (4.884,6.311) | 0.91 (0.759,1.062) |
| Antigua and Barbuda | 0.001 (0.001,0.001) | 1.168 (1.018,1.338) | 0.002 (0.002,0.002) | 1.742 (1.584,1.923) | 1.534 (1.45,1.618) |
| Barbados | 0.005 (0.004,0.005) | 1.767 (1.594,1.963) | 0.012 (0.011,0.013) | 2.539 (2.346,2.733) | 1.414 (1.305,1.522) |
| Cuba | 0.095 (0.079,0.112) | 0.891 (0.748,1.046) | 0.152 (0.134,0.173) | 0.935 (0.818,1.065) | 0.168 (0.077,0.259) |
| Dominica | 0.001 (0.001,0.001) | 1.583 (1.401,1.789) | 0.002 (0.002,0.002) | 2.308 (2.108,2.509) | 1.389 (1.304,1.474) |
| Dominican Republic | 0.047 (0.039,0.056) | 1.007 (0.857,1.16) | 0.148 (0.132,0.166) | 1.401 (1.253,1.564) | 1.354 (1.22,1.488) |
| Grenada | 0.001 (0.001,0.001) | 1.596 (1.426,1.782) | 0.003 (0.002,0.003) | 2.32 (2.141,2.516) | 1.317 (1.202,1.432) |
| Jamaica | 0.02 (0.017,0.023) | 1.083 (0.948,1.235) | 0.047 (0.043,0.052) | 1.492 (1.356,1.644) | 1.106 (0.979,1.233) |
| Trinidad and Tobago | 0.022 (0.02,0.024) | 2.512 (2.298,2.745) | 0.057 (0.052,0.061) | 3.063 (2.846,3.307) | 1.096 (0.898,1.294) |
| Costa Rica | 0.093 (0.085,0.101) | 4.984 (4.601,5.432) | 0.392 (0.366,0.42) | 7.084 (6.617,7.558) | 1.088 (0.919,1.258) |
| El Salvador | 0.091 (0.082,0.101) | 2.881 (2.605,3.164) | 0.309 (0.283,0.335) | 4.898 (4.497,5.31) | 2.212 (2.008,2.417) |
| Honduras | 0.077 (0.068,0.087) | 3.389 (2.997,3.79) | 0.363 (0.329,0.4) | 5.307 (4.806,5.806) | 1.575 (1.491,1.658) |
| Nicaragua | 0.039 (0.033,0.044) | 2.137 (1.884,2.419) | 0.184 (0.168,0.201) | 3.638 (3.315,3.975) | 2.137 (1.938,2.336) |
| Panama | 0.042 (0.038,0.047) | 2.574 (2.332,2.837) | 0.216 (0.2,0.232) | 4.854 (4.497,5.222) | 2.387 (2.288,2.487) |

## Table S2 The mortality number, age-standardized rates and temporal trends of Interstitial lung diseases in the Belt and Road Initiative countries from 1990 to 2021.

| **location** |  | | | | |
| --- | --- | --- | --- | --- | --- |
|  | **Number of cases, 1990  (thousands)** | **Age-standardised rate per 100,000 population, 1990** | **Number of cases, 2021  (thousands)** | **Age-standardised rate per 100,000 population, 2021** | **Estimated annual percentage change, 1990-2021** |
| Global | 54.967 (44.761,68.391) | 1.517 (1.251,1.868) | 188.222 (161.406,212.252) | 2.28 (1.959,2.563) | 1.553 (1.41,1.697) |
| Female | 23.785 (18.18,32.747) | 1.168 (0.902,1.599) | 85.166 (68.72,105.539) | 1.827 (1.475,2.265) | 1.698 (1.533,1.864) |
| Male | 31.183 (24.196,39.012) | 2.01 (1.606,2.481) | 103.057 (84.156,115.833) | 2.899 (2.397,3.238) | 1.407 (1.279,1.535) |
| High SDI | 20.064 (18.622,20.871) | 1.79 (1.66,1.863) | 81.732 (71.244,88.092) | 3.443 (3.046,3.693) | 2.302 (2.045,2.559) |
| High-middle SDI | 8.238 (7.611,9.227) | 0.91 (0.837,1.019) | 22.851 (20.008,25.163) | 1.192 (1.041,1.313) | 1.237 (1.081,1.393) |
| Middle SDI | 8.993 (7.004,12.322) | 1.072 (0.85,1.453) | 33.342 (27.822,40.427) | 1.388 (1.155,1.68) | 1.15 (1.025,1.275) |
| Low-middle SDI | 13.153 (7.523,20.491) | 2.483 (1.455,3.808) | 39.118 (26.54,53.612) | 3.085 (2.124,4.228) | 0.943 (0.825,1.061) |
| Low SDI | 4.478 (2.158,6.438) | 2.342 (1.192,3.266) | 11.084 (6.589,15.794) | 2.61 (1.558,3.746) | 0.632 (0.445,0.82) |
| High-income North America | 8.066 (7.442,8.421) | 2.233 (2.068,2.328) | 29.737 (26.085,31.529) | 4.246 (3.752,4.486) | 2.164 (1.797,2.533) |
| Caribbean | 0.175 (0.141,0.219) | 0.696 (0.573,0.86) | 0.639 (0.534,0.773) | 1.186 (0.991,1.447) | 1.993 (1.773,2.213) |
| Andean Latin America | 1.444 (1.045,2.078) | 7.859 (5.682,11.331) | 6.366 (4.875,8.022) | 11.374 (8.687,14.332) | 1.873 (1.683,2.062) |
| Central Latin America | 1.12 (1.064,1.191) | 1.445 (1.361,1.54) | 6.475 (5.837,7.126) | 2.687 (2.423,2.958) | 2.188 (1.963,2.414) |
| Tropical Latin America | 0.847 (0.799,0.89) | 0.98 (0.905,1.044) | 4.527 (4.08,4.836) | 1.83 (1.645,1.959) | 2.13 (1.79,2.472) |
| North Africa and Middle East | 0.919 (0.633,1.433) | 0.64 (0.44,1.004) | 3.051 (2.249,4.527) | 0.763 (0.561,1.152) | 0.872 (0.672,1.072) |
| South Asia | 17.522 (9.598,27.913) | 3.616 (2.004,5.648) | 54.724 (35.863,74.243) | 4.192 (2.812,5.764) | 0.668 (0.536,0.8) |
| Central Sub-Saharan Africa | 0.259 (0.088,0.549) | 1.417 (0.487,3.368) | 0.666 (0.242,1.622) | 1.472 (0.508,3.917) | 0.091 (-0.017,0.199) |
| Eastern Sub-Saharan Africa | 0.687 (0.228,1.206) | 1.025 (0.352,1.934) | 1.484 (0.53,3.098) | 0.961 (0.333,2.082) | -0.317 (-0.375,-0.259) |
| Southern Sub-Saharan Africa | 0.493 (0.262,0.752) | 2.081 (1.075,3.246) | 0.986 (0.677,1.309) | 1.995 (1.39,2.649) | -0.339 (-0.592,-0.085) |
| Western Sub-Saharan Africa | 1.332 (0.486,2.109) | 1.727 (0.655,2.771) | 2.386 (0.919,4.115) | 1.389 (0.54,2.412) | -0.638 (-0.76,-0.516) |
| Oceania | 0.071 (0.048,0.11) | 2.16 (1.448,3.464) | 0.185 (0.119,0.304) | 2.298 (1.416,3.847) | 0.231 (0.163,0.298) |
| Central Asia | 0.715 (0.616,0.821) | 1.639 (1.378,1.91) | 0.63 (0.515,0.779) | 0.866 (0.711,1.061) | -2.054 (-2.509,-1.597) |
| Central Europe | 1.513 (1.42,1.622) | 1.043 (0.982,1.118) | 2.179 (1.973,2.369) | 0.974 (0.884,1.059) | 0.121 (-0.222,0.465) |
| East Asia | 3.042 (2.259,4.831) | 0.407 (0.309,0.657) | 8.19 (5.177,10.927) | 0.405 (0.255,0.538) | 0.383 (0.19,0.576) |
| Eastern Europe | 2.86 (2.66,3.06) | 1.101 (1.021,1.177) | 1.154 (1.054,1.263) | 0.335 (0.307,0.367) | -5.474 (-6.401,-4.538) |
| High-income Asia Pacific | 5.196 (4.709,5.608) | 2.718 (2.45,2.936) | 26.166 (21.826,28.826) | 4.356 (3.726,4.761) | 1.346 (1.107,1.586) |
| Australasia | 0.263 (0.242,0.285) | 1.131 (1.03,1.223) | 1.904 (1.603,2.088) | 3.17 (2.686,3.456) | 3.545 (2.979,4.114) |
| Western Europe | 6.645 (6.204,6.944) | 1.109 (1.035,1.158) | 30.538 (26.845,32.788) | 2.787 (2.493,2.972) | 3.742 (3.377,4.108) |
| Southeast Asia | 0.659 (0.317,1.48) | 0.292 (0.143,0.655) | 1.877 (0.959,3.772) | 0.33 (0.17,0.655) | 0.422 (0.341,0.504) |
| Southern Latin America | 1.14 (1.066,1.223) | 2.579 (2.408,2.767) | 4.357 (3.906,4.681) | 4.796 (4.313,5.146) | 2.293 (1.902,2.686) |
| China | 2.955 (2.186,4.688) | 0.41 (0.31,0.665) | 7.674 (4.637,10.371) | 0.393 (0.237,0.531) | 0.239 (0.054,0.425) |
| Armenia | 0.055 (0.05,0.06) | 2.138 (1.935,2.321) | 0.057 (0.05,0.065) | 1.344 (1.172,1.526) | 0.019 (-1.13,1.182) |
| Azerbaijan | 0.061 (0.029,0.114) | 1.352 (0.608,2.63) | 0.072 (0.033,0.129) | 0.805 (0.378,1.423) | -1.845 (-2.257,-1.431) |
| Georgia | 0.035 (0.029,0.043) | 0.61 (0.497,0.744) | 0.035 (0.03,0.041) | 0.572 (0.487,0.667) | 1.827 (0.707,2.959) |
| Kazakhstan | 0.106 (0.093,0.121) | 0.892 (0.771,1.017) | 0.143 (0.108,0.184) | 0.854 (0.644,1.122) | -0.152 (-0.847,0.548) |
| Kyrgyzstan | 0.031 (0.026,0.036) | 1.084 (0.901,1.286) | 0.01 (0.008,0.012) | 0.229 (0.186,0.281) | -5.738 (-6.909,-4.552) |
| Mongolia | 0.026 (0.016,0.04) | 2.542 (1.518,3.889) | 0.03 (0.018,0.046) | 1.512 (0.907,2.464) | -2.193 (-2.446,-1.938) |
| Tajikistan | 0.107 (0.053,0.189) | 4.332 (2.055,7.91) | 0.131 (0.07,0.24) | 2.925 (1.592,5.216) | -1.549 (-1.756,-1.342) |
| Turkmenistan | 0.023 (0.02,0.026) | 1.272 (1.063,1.412) | 0.021 (0.017,0.028) | 0.515 (0.399,0.664) | -3.577 (-4.332,-2.816) |
| Uzbekistan | 0.269 (0.208,0.357) | 2.445 (1.841,3.336) | 0.131 (0.11,0.157) | 0.577 (0.487,0.689) | -5.153 (-5.975,-4.324) |
| Brunei Darussalam | 0.003 (0.002,0.004) | 3.205 (2.073,4.608) | 0.007 (0.005,0.009) | 2.496 (1.624,3.463) | -0.58 (-0.734,-0.427) |
| Republic of Korea | 0.473 (0.285,0.838) | 1.999 (1.18,3.568) | 2.052 (1.11,2.823) | 2.183 (1.183,2.992) | 0.784 (0.593,0.975) |
| Singapore | 0.017 (0.016,0.019) | 0.899 (0.827,0.967) | 0.082 (0.071,0.089) | 1 (0.867,1.095) | 0.756 (0.506,1.007) |
| Afghanistan | 0.004 (0,0.015) | 0.065 (0.001,0.246) | 0.022 (0.002,0.08) | 0.262 (0.022,0.859) | 5.901 (5.288,6.518) |
| Bahrain | 0.003 (0.002,0.005) | 2.435 (1.709,4.339) | 0.017 (0.01,0.025) | 3.157 (1.91,4.534) | 1.063 (0.683,1.445) |
| Iran (Islamic Republic of) | 0.004 (0.001,0.008) | 0.02 (0.006,0.038) | 0.032 (0.005,0.059) | 0.046 (0.007,0.086) | 3.832 (3.28,4.388) |
| Iraq | 0.05 (0.033,0.076) | 0.637 (0.412,1) | 0.159 (0.099,0.23) | 0.781 (0.498,1.16) | 0.512 (0.357,0.667) |
| Jordan | 0.028 (0.018,0.048) | 2.399 (1.517,4.067) | 0.134 (0.091,0.186) | 2.237 (1.506,3.142) | -0.132 (-0.305,0.042) |
| Kuwait | 0.014 (0.012,0.015) | 2.724 (2.385,3.019) | 0.052 (0.042,0.06) | 2.22 (1.789,2.605) | 0.527 (-0.549,1.616) |
| Lebanon | 0.036 (0.017,0.068) | 1.894 (0.901,3.52) | 0.122 (0.087,0.181) | 1.877 (1.35,2.79) | 0.381 (0.202,0.56) |
| Oman | 0.003 (0.002,0.006) | 0.552 (0.326,0.971) | 0.013 (0.007,0.021) | 0.805 (0.418,1.263) | 2.089 (1.615,2.565) |
| Palestine | 0.032 (0.022,0.044) | 4.209 (2.869,5.789) | 0.08 (0.051,0.106) | 4.077 (2.524,5.456) | 0.034 (-0.09,0.159) |
| Qatar | 0.001 (0.001,0.001) | 1.291 (0.913,2.116) | 0.006 (0.003,0.009) | 0.976 (0.61,1.462) | -0.794 (-1.128,-0.459) |
| Saudi Arabia | 0.217 (0.137,0.369) | 4.444 (2.816,7.809) | 0.77 (0.511,1.084) | 5.306 (3.272,7.595) | 0.918 (0.704,1.132) |
| Syrian Arab Republic | 0.033 (0.021,0.054) | 0.713 (0.438,1.138) | 0.104 (0.063,0.17) | 1.019 (0.622,1.64) | 1.277 (1.122,1.432) |
| Turkey | 0.3 (0.173,0.501) | 0.959 (0.546,1.618) | 0.976 (0.645,1.43) | 1.115 (0.738,1.655) | 0.918 (0.624,1.214) |
| United Arab Emirates | 0.022 (0.013,0.036) | 5.33 (3.212,8.753) | 0.118 (0.079,0.17) | 5.29 (3.696,7.502) | 2.054 (1.367,2.746) |
| Yemen | 0.002 (0,0.008) | 0.046 (0.001,0.16) | 0.025 (0.002,0.081) | 0.21 (0.02,0.673) | 6.318 (5.672,6.968) |
| Bangladesh | 1.374 (0.748,2.19) | 3.132 (1.682,4.996) | 3.505 (2.283,5.283) | 2.718 (1.775,4.08) | -0.584 (-0.761,-0.408) |
| Nepal | 0.372 (0.189,0.631) | 4.757 (2.466,7.776) | 1.118 (0.722,1.594) | 5.409 (3.486,7.696) | 0.573 (0.401,0.744) |
| Pakistan | 1.298 (0.782,1.912) | 2.596 (1.557,3.841) | 2.747 (1.805,3.841) | 2.773 (1.833,3.925) | 0.07 (-0.082,0.222) |
| Cambodia | 0.006 (0.001,0.021) | 0.16 (0.025,0.523) | 0.019 (0.004,0.058) | 0.189 (0.037,0.575) | 0.649 (0.566,0.732) |
| Indonesia | 0.195 (0.034,0.594) | 0.222 (0.038,0.676) | 0.535 (0.113,1.497) | 0.264 (0.054,0.746) | 0.578 (0.512,0.644) |
| Lao People's Democratic Republic | 0.004 (0,0.015) | 0.249 (0.029,0.796) | 0.01 (0.002,0.029) | 0.238 (0.04,0.745) | -0.175 (-0.201,-0.15) |
| Malaysia | 0.07 (0.052,0.103) | 0.791 (0.585,1.176) | 0.24 (0.168,0.323) | 0.927 (0.647,1.253) | 0.621 (0.399,0.843) |
| Maldives | 0.005 (0.002,0.008) | 6.091 (3.171,10.625) | 0.011 (0.007,0.015) | 3.475 (2.41,4.769) | -1.962 (-2.058,-1.865) |
| Myanmar | 0.078 (0.01,0.276) | 0.405 (0.055,1.382) | 0.197 (0.037,0.597) | 0.478 (0.089,1.468) | 0.506 (0.458,0.555) |
| Philippines | 0.011 (0.007,0.017) | 0.048 (0.031,0.075) | 0.036 (0.021,0.048) | 0.048 (0.028,0.065) | -0.007 (-0.185,0.171) |
| Sri Lanka | 0.118 (0.09,0.169) | 1.255 (0.95,1.831) | 0.27 (0.152,0.439) | 1.095 (0.619,1.754) | -0.258 (-0.478,-0.038) |
| Thailand | 0.076 (0.054,0.109) | 0.229 (0.163,0.335) | 0.189 (0.118,0.319) | 0.18 (0.113,0.3) | -1.139 (-1.281,-0.996) |
| Timor-Leste | 0.001 (0,0.002) | 0.205 (0.032,0.595) | 0.002 (0,0.005) | 0.213 (0.036,0.603) | 0.296 (0.1,0.493) |
| Viet Nam | 0.075 (0.013,0.221) | 0.206 (0.036,0.602) | 0.203 (0.037,0.565) | 0.24 (0.044,0.676) | 0.643 (0.559,0.726) |
| Albania | 0.022 (0.014,0.034) | 1.182 (0.748,1.795) | 0.036 (0.019,0.062) | 0.839 (0.434,1.442) | -1.121 (-1.313,-0.929) |
| Bosnia and Herzegovina | 0.031 (0.018,0.049) | 0.825 (0.487,1.306) | 0.034 (0.019,0.056) | 0.543 (0.307,0.908) | -1.599 (-1.788,-1.409) |
| Bulgaria | 0.048 (0.042,0.055) | 0.426 (0.377,0.479) | 0.067 (0.056,0.08) | 0.483 (0.401,0.574) | 0.416 (0.111,0.722) |
| Croatia | 0.016 (0.015,0.017) | 0.278 (0.259,0.3) | 0.031 (0.027,0.035) | 0.333 (0.29,0.376) | 1.164 (0.875,1.453) |
| Czechia | 0.095 (0.084,0.11) | 0.713 (0.63,0.814) | 0.355 (0.305,0.41) | 1.563 (1.344,1.802) | 3.635 (3.287,3.985) |
| Hungary | 0.135 (0.126,0.145) | 0.946 (0.882,1.014) | 0.246 (0.214,0.278) | 1.254 (1.09,1.42) | 1.619 (1.252,1.987) |
| North Macedonia | 0.004 (0.003,0.005) | 0.205 (0.147,0.289) | 0.007 (0.003,0.014) | 0.242 (0.116,0.451) | 0.521 (0.257,0.786) |
| Montenegro | 0.001 (0,0.001) | 0.111 (0.063,0.189) | 0.001 (0.001,0.002) | 0.11 (0.062,0.179) | 0.002 (-0.209,0.213) |
| Poland | 0.352 (0.338,0.364) | 0.814 (0.78,0.841) | 0.749 (0.683,0.814) | 1.037 (0.946,1.125) | 1.124 (0.562,1.689) |
| Romania | 0.675 (0.596,0.772) | 2.578 (2.286,2.91) | 0.424 (0.371,0.481) | 1.164 (1.012,1.322) | -2.452 (-2.858,-2.045) |
| Serbia | 0.053 (0.037,0.076) | 0.554 (0.388,0.802) | 0.075 (0.043,0.109) | 0.448 (0.26,0.654) | -0.611 (-0.713,-0.509) |
| Slovakia | 0.034 (0.024,0.05) | 0.571 (0.412,0.849) | 0.055 (0.029,0.086) | 0.577 (0.312,0.902) | 0.366 (0.231,0.5) |
| Slovenia | 0.023 (0.021,0.025) | 0.93 (0.844,1.026) | 0.068 (0.057,0.078) | 1.412 (1.183,1.622) | 1.584 (1.285,1.883) |
| Belarus | 0.233 (0.2,0.264) | 1.928 (1.653,2.197) | 0.042 (0.034,0.05) | 0.265 (0.216,0.317) | -7.607 (-8.205,-7.005) |
| Estonia | 0.055 (0.049,0.062) | 2.759 (2.459,3.103) | 0.009 (0.008,0.011) | 0.301 (0.256,0.348) | -8.828 (-10.982,-6.623) |
| Latvia | 0.136 (0.122,0.152) | 3.839 (3.452,4.252) | 0.014 (0.012,0.016) | 0.31 (0.263,0.36) | -9.224 (-11.282,-7.119) |
| Lithuania | 0.066 (0.057,0.077) | 1.476 (1.286,1.718) | 0.01 (0.009,0.011) | 0.159 (0.138,0.181) | -8.593 (-11.133,-5.98) |
| Republic of Moldova | 0.022 (0.02,0.025) | 0.592 (0.525,0.667) | 0.002 (0.002,0.003) | 0.039 (0.034,0.045) | -10.078 (-12.19,-7.915) |
| Russian Federation | 1.489 (1.305,1.64) | 0.896 (0.786,0.987) | 0.858 (0.794,0.925) | 0.364 (0.337,0.393) | -4.759 (-6.055,-3.444) |
| Ukraine | 0.859 (0.769,0.941) | 1.236 (1.109,1.351) | 0.219 (0.163,0.283) | 0.296 (0.22,0.383) | -5.926 (-6.412,-5.438) |
| Austria | 0.067 (0.062,0.072) | 0.535 (0.499,0.575) | 0.249 (0.216,0.276) | 1.22 (1.073,1.341) | 3.785 (3.463,4.108) |
| Cyprus | 0.048 (0.031,0.076) | 8.026 (5.224,12.299) | 0.089 (0.06,0.123) | 4.464 (3.009,6.143) | -1.722 (-2.022,-1.42) |
| Greece | 0.061 (0.056,0.067) | 0.417 (0.382,0.451) | 0.546 (0.479,0.598) | 1.951 (1.76,2.117) | 5.978 (5.195,6.767) |
| Italy | 0.191 (0.178,0.199) | 0.214 (0.201,0.224) | 3.277 (2.813,3.568) | 1.938 (1.706,2.093) | 8.564 (7.043,10.107) |
| Luxembourg | 0.004 (0.004,0.004) | 0.738 (0.678,0.8) | 0.021 (0.018,0.023) | 1.813 (1.596,2.033) | 3.717 (3.257,4.18) |
| Malta | 0.008 (0.007,0.008) | 1.852 (1.673,2.029) | 0.048 (0.041,0.054) | 4.379 (3.762,4.964) | 3.098 (2.616,3.583) |
| Portugal | 0.107 (0.099,0.115) | 0.813 (0.751,0.877) | 0.724 (0.615,0.8) | 2.5 (2.167,2.743) | 4.483 (3.8,5.169) |
| Algeria | 0.004 (0,0.013) | 0.042 (0.001,0.138) | 0.059 (0.005,0.218) | 0.208 (0.018,0.725) | 6.881 (6.175,7.591) |
| Egypt | 0.154 (0.111,0.223) | 0.697 (0.505,1.016) | 0.22 (0.154,0.312) | 0.457 (0.321,0.631) | -1.658 (-1.958,-1.357) |
| Libya | 0.001 (0,0.003) | 0.045 (0.001,0.163) | 0.014 (0.001,0.056) | 0.305 (0.021,1.197) | 8.206 (7.513,8.905) |
| Morocco | 0.005 (0,0.017) | 0.038 (0.001,0.126) | 0.064 (0.006,0.2) | 0.211 (0.021,0.668) | 7.166 (6.475,7.861) |
| Tunisia | 0.002 (0,0.005) | 0.037 (0.001,0.12) | 0.022 (0.002,0.085) | 0.183 (0.014,0.696) | 6.548 (5.94,7.159) |
| Angola | 0.04 (0.013,0.079) | 1.222 (0.432,2.646) | 0.103 (0.038,0.192) | 1.06 (0.4,2.1) | -0.629 (-0.684,-0.574) |
| Central African Republic | 0.014 (0.004,0.027) | 1.527 (0.504,3.292) | 0.026 (0.009,0.053) | 1.431 (0.534,3.418) | -0.246 (-0.314,-0.178) |
| Congo | 0.014 (0.004,0.031) | 1.624 (0.531,3.743) | 0.03 (0.012,0.058) | 1.345 (0.511,2.719) | -0.77 (-0.849,-0.691) |
| Democratic Republic of the Congo | 0.181 (0.061,0.41) | 1.441 (0.47,3.675) | 0.492 (0.17,1.335) | 1.624 (0.52,4.851) | 0.4 (0.257,0.542) |
| Equatorial Guinea | 0.002 (0.001,0.005) | 1.42 (0.486,2.876) | 0.005 (0.002,0.011) | 1.164 (0.404,2.496) | -0.745 (-0.803,-0.688) |
| Gabon | 0.007 (0.003,0.014) | 1.468 (0.514,2.898) | 0.01 (0.004,0.022) | 1.153 (0.418,2.534) | -0.885 (-0.955,-0.816) |
| Burundi | 0.027 (0.008,0.049) | 1.235 (0.398,2.256) | 0.044 (0.015,0.094) | 1.027 (0.337,2.259) | -0.829 (-0.991,-0.666) |
| Comoros | 0.002 (0.001,0.004) | 1.149 (0.357,2.303) | 0.004 (0.001,0.009) | 0.901 (0.322,1.973) | -0.943 (-1.163,-0.722) |
| Djibouti | 0.001 (0,0.002) | 0.951 (0.334,1.748) | 0.004 (0.001,0.009) | 0.751 (0.264,1.547) | -0.86 (-0.992,-0.729) |
| Eritrea | 0.011 (0.003,0.022) | 1.127 (0.35,1.975) | 0.025 (0.009,0.043) | 1.02 (0.397,1.793) | -0.399 (-0.489,-0.31) |
| Ethiopia | 0.164 (0.043,0.292) | 0.902 (0.264,1.616) | 0.307 (0.096,0.654) | 0.757 (0.228,1.651) | -0.816 (-0.912,-0.719) |
| Kenya | 0.072 (0.019,0.206) | 0.973 (0.238,2.942) | 0.24 (0.062,0.758) | 1.221 (0.308,4.13) | 0.838 (0.772,0.904) |
| Madagascar | 0.075 (0.027,0.134) | 1.624 (0.587,3.058) | 0.165 (0.062,0.331) | 1.715 (0.626,3.537) | 0.08 (0.03,0.13) |
| Malawi | 0.03 (0.009,0.055) | 0.867 (0.285,1.659) | 0.067 (0.025,0.134) | 0.985 (0.362,2.027) | 0.302 (0.218,0.385) |
| Mozambique | 0.038 (0.012,0.072) | 0.712 (0.243,1.404) | 0.081 (0.029,0.159) | 0.793 (0.306,1.626) | 0.468 (0.359,0.578) |
| Rwanda | 0.034 (0.01,0.06) | 1.293 (0.428,2.233) | 0.056 (0.019,0.128) | 0.992 (0.328,2.33) | -1.332 (-1.555,-1.109) |
| Seychelles | 0 (0,0) | 0.202 (0.04,0.596) | 0 (0,0.001) | 0.172 (0.032,0.507) | -0.434 (-0.543,-0.325) |
| Somalia | 0.025 (0.007,0.048) | 1.236 (0.374,2.29) | 0.055 (0.019,0.102) | 0.984 (0.369,1.852) | -0.71 (-0.783,-0.637) |
| United Republic of Tanzania | 0.081 (0.027,0.149) | 0.81 (0.269,1.602) | 0.187 (0.063,0.42) | 0.782 (0.261,1.749) | -0.228 (-0.276,-0.18) |
| Uganda | 0.069 (0.022,0.14) | 1.173 (0.372,2.474) | 0.143 (0.047,0.341) | 1.047 (0.344,2.599) | -0.612 (-0.709,-0.515) |
| Zambia | 0.025 (0.008,0.043) | 0.938 (0.334,1.696) | 0.068 (0.025,0.136) | 1.048 (0.383,2.13) | 0.367 (0.28,0.454) |
| Botswana | 0.009 (0.002,0.019) | 2.136 (0.504,4.529) | 0.017 (0.004,0.036) | 1.437 (0.359,3.083) | -1.213 (-1.361,-1.065) |
| Lesotho | 0.012 (0.003,0.024) | 1.603 (0.364,3.28) | 0.015 (0.004,0.031) | 1.554 (0.385,3.132) | 0.196 (0.03,0.363) |
| Namibia | 0.011 (0.003,0.022) | 2.074 (0.485,4.509) | 0.021 (0.005,0.045) | 1.86 (0.466,3.842) | -0.449 (-0.541,-0.356) |
| South Africa | 0.439 (0.247,0.654) | 2.35 (1.287,3.593) | 0.894 (0.649,1.266) | 2.194 (1.608,3.1) | -0.491 (-0.773,-0.207) |
| Zimbabwe | 0.017 (0.003,0.032) | 0.527 (0.098,1.004) | 0.031 (0.006,0.063) | 0.561 (0.115,1.139) | 0.285 (0.144,0.426) |
| Benin | 0.038 (0.014,0.065) | 2.023 (0.726,3.587) | 0.064 (0.023,0.138) | 1.372 (0.488,3.028) | -1.194 (-1.456,-0.931) |
| Burkina Faso | 0.047 (0.015,0.079) | 1.25 (0.424,2.188) | 0.08 (0.03,0.15) | 0.952 (0.363,1.853) | -0.944 (-1.076,-0.812) |
| Cameroon | 0.091 (0.034,0.155) | 2.374 (0.905,4.181) | 0.178 (0.066,0.332) | 1.566 (0.589,2.97) | -1.27 (-1.507,-1.033) |
| Cabo Verde | 0.006 (0.002,0.013) | 2.552 (0.684,5.424) | 0.005 (0.002,0.009) | 1.107 (0.418,2.065) | -2.449 (-3.17,-1.723) |
| Chad | 0.048 (0.016,0.087) | 1.851 (0.652,3.275) | 0.08 (0.032,0.15) | 1.544 (0.605,3.005) | -0.472 (-0.672,-0.271) |
| Côte d'Ivoire | 0.071 (0.025,0.122) | 2.166 (0.783,3.835) | 0.142 (0.049,0.277) | 1.455 (0.516,2.942) | -1.201 (-1.439,-0.963) |
| Gambia | 0.007 (0.002,0.012) | 2.119 (0.73,3.909) | 0.015 (0.006,0.031) | 1.673 (0.65,3.499) | -0.716 (-1.018,-0.414) |
| Ghana | 0.077 (0.027,0.126) | 1.442 (0.514,2.382) | 0.213 (0.087,0.358) | 1.469 (0.596,2.425) | 0.227 (0.129,0.325) |
| Guinea | 0.058 (0.022,0.099) | 1.927 (0.726,3.377) | 0.079 (0.031,0.163) | 1.532 (0.576,3.294) | -0.577 (-0.79,-0.364) |
| Guinea-Bissau | 0.009 (0.003,0.017) | 2.478 (0.834,4.383) | 0.011 (0.004,0.019) | 1.703 (0.716,3.033) | -1.049 (-1.305,-0.793) |
| Liberia | 0.021 (0.007,0.039) | 2.035 (0.699,3.866) | 0.029 (0.01,0.062) | 1.533 (0.506,3.502) | -0.805 (-1.086,-0.523) |
| Mali | 0.098 (0.031,0.167) | 2.81 (0.937,4.932) | 0.196 (0.068,0.408) | 2.441 (0.828,5.171) | -0.387 (-0.49,-0.283) |
| Mauritania | 0.019 (0.007,0.032) | 2.089 (0.753,3.509) | 0.026 (0.01,0.049) | 1.326 (0.497,2.578) | -1.474 (-1.841,-1.107) |
| Niger | 0.048 (0.016,0.085) | 2.034 (0.717,3.581) | 0.102 (0.038,0.207) | 1.466 (0.531,3.096) | -0.875 (-1.135,-0.614) |
| Nigeria | 0.577 (0.21,0.942) | 1.493 (0.554,2.436) | 0.96 (0.35,1.807) | 1.238 (0.452,2.311) | -0.611 (-0.662,-0.559) |
| Sao Tome and Principe | 0.003 (0.001,0.005) | 4.318 (1.471,7.962) | 0.004 (0.001,0.008) | 3.827 (1.437,8.033) | -0.361 (-0.428,-0.295) |
| Senegal | 0.059 (0.02,0.1) | 2.046 (0.718,3.511) | 0.105 (0.04,0.21) | 1.511 (0.563,3.041) | -0.801 (-1.181,-0.419) |
| Sierra Leone | 0.035 (0.012,0.063) | 1.888 (0.641,3.452) | 0.047 (0.017,0.099) | 1.377 (0.486,2.962) | -0.873 (-1.136,-0.609) |
| Togo | 0.022 (0.008,0.038) | 1.998 (0.734,3.617) | 0.051 (0.019,0.107) | 1.557 (0.567,3.377) | -0.732 (-0.964,-0.499) |
| South Sudan | 0.03 (0.009,0.061) | 1.225 (0.401,2.551) | 0.037 (0.014,0.078) | 1.08 (0.406,2.379) | -0.535 (-0.73,-0.339) |
| Sudan | 0.005 (0,0.018) | 0.053 (0.001,0.2) | 0.04 (0.003,0.139) | 0.226 (0.018,0.796) | 6.033 (5.448,6.622) |
| Guyana | 0.005 (0.005,0.006) | 1.462 (1.282,1.64) | 0.01 (0.008,0.013) | 1.753 (1.344,2.217) | 1.824 (0.991,2.663) |
| Suriname | 0.003 (0.002,0.004) | 1.058 (0.778,1.639) | 0.008 (0.004,0.011) | 1.266 (0.722,1.834) | 1.146 (0.949,1.344) |
| Bolivia (Plurinational State of) | 0.217 (0.104,0.429) | 8.23 (4.085,15.863) | 0.73 (0.458,1.069) | 9.743 (6.253,14.08) | 0.817 (0.712,0.922) |
| Ecuador | 0.15 (0.134,0.168) | 3.274 (2.91,3.656) | 1.209 (0.996,1.471) | 8.107 (6.714,9.782) | 4.087 (3.553,4.625) |
| Peru | 1.077 (0.773,1.519) | 9.75 (6.887,13.643) | 4.428 (3.073,5.829) | 13.315 (9.198,17.552) | 1.65 (1.419,1.881) |
| Venezuela (Bolivarian Republic of) | 0.081 (0.074,0.088) | 0.872 (0.789,0.954) | 0.425 (0.327,0.552) | 1.508 (1.172,1.952) | 2.216 (1.928,2.505) |
| Brazil | 0.827 (0.781,0.874) | 0.981 (0.907,1.049) | 4.453 (4.017,4.763) | 1.841 (1.657,1.973) | 2.138 (1.79,2.487) |
| Argentina | 0.731 (0.663,0.8) | 2.366 (2.142,2.589) | 2.001 (1.785,2.186) | 3.445 (3.077,3.757) | 1.574 (1.11,2.041) |
| Chile | 0.356 (0.333,0.379) | 3.834 (3.561,4.102) | 2.169 (1.943,2.336) | 8.252 (7.407,8.884) | 2.679 (2.296,3.064) |
| Uruguay | 0.053 (0.048,0.058) | 1.364 (1.236,1.485) | 0.186 (0.164,0.204) | 2.909 (2.615,3.164) | 2.804 (2.525,3.084) |
| Fiji | 0.003 (0.002,0.004) | 0.652 (0.458,0.889) | 0.005 (0.003,0.007) | 0.694 (0.414,0.984) | 0.294 (0.192,0.396) |
| Kiribati | 0.001 (0,0.001) | 1.728 (0.85,3.039) | 0.001 (0.001,0.002) | 1.742 (0.906,3.1) | -0.042 (-0.076,-0.008) |
| Micronesia (Federated States of) | 0.001 (0.001,0.002) | 2.013 (1.124,3.408) | 0.001 (0.001,0.002) | 1.668 (0.93,3.002) | -0.596 (-0.682,-0.509) |
| Papua New Guinea | 0.051 (0.031,0.081) | 2.569 (1.559,4.322) | 0.151 (0.092,0.253) | 2.796 (1.589,4.839) | 0.308 (0.233,0.383) |
| Samoa | 0.002 (0.001,0.003) | 1.9 (1.134,3.271) | 0.003 (0.001,0.005) | 1.71 (0.99,3.131) | -0.263 (-0.337,-0.188) |
| Solomon Islands | 0.001 (0.001,0.002) | 1.016 (0.652,1.651) | 0.004 (0.002,0.007) | 1.08 (0.677,1.906) | 0.259 (0.16,0.358) |
| Tonga | 0.001 (0,0.001) | 1.222 (0.798,2.051) | 0.001 (0.001,0.002) | 1.217 (0.705,2.249) | 0.161 (0.089,0.233) |
| Vanuatu | 0.001 (0.001,0.002) | 1.812 (0.939,2.983) | 0.004 (0.002,0.006) | 1.775 (0.912,3.228) | -0.218 (-0.303,-0.133) |
| Cook Islands | 0 (0,0) | 1.293 (0.891,2.192) | 0 (0,0) | 0.906 (0.452,1.783) | -1.289 (-1.404,-1.175) |
| Nauru | 0 (0,0) | 1.978 (1.068,3.665) | 0 (0,0) | 1.91 (0.958,3.772) | -0.13 (-0.18,-0.08) |
| Niue | 0 (0,0) | 1.773 (1.202,2.864) | 0 (0,0) | 1.873 (1.166,3.061) | -0.481 (-0.728,-0.233) |
| New Zealand | 0.049 (0.044,0.053) | 1.221 (1.118,1.322) | 0.271 (0.236,0.299) | 2.971 (2.597,3.263) | 2.923 (2.669,3.178) |
| Antigua and Barbuda | 0 (0,0) | 0.458 (0.41,0.51) | 0.001 (0.001,0.001) | 1.128 (1.035,1.235) | 3.519 (3.169,3.871) |
| Barbados | 0.003 (0.002,0.003) | 0.895 (0.81,0.977) | 0.01 (0.008,0.012) | 1.898 (1.546,2.299) | 3.273 (2.904,3.644) |
| Cuba | 0.017 (0.016,0.019) | 0.171 (0.158,0.186) | 0.065 (0.057,0.074) | 0.331 (0.288,0.375) | 2.669 (2.35,2.989) |
| Dominica | 0 (0,0.001) | 0.766 (0.565,1.176) | 0.001 (0.001,0.001) | 1.151 (0.722,1.627) | 1.455 (1.301,1.608) |
| Dominican Republic | 0.012 (0.007,0.023) | 0.34 (0.197,0.711) | 0.047 (0.025,0.084) | 0.48 (0.252,0.844) | 1.476 (1.226,1.726) |
| Grenada | 0.001 (0,0.001) | 0.681 (0.578,0.784) | 0.002 (0.001,0.002) | 1.685 (1.451,1.907) | 3.504 (3.088,3.921) |
| Jamaica | 0.007 (0.006,0.008) | 0.381 (0.345,0.42) | 0.033 (0.025,0.041) | 1.029 (0.787,1.303) | 3.802 (3.395,4.21) |
| Trinidad and Tobago | 0.012 (0.011,0.013) | 1.589 (1.44,1.746) | 0.046 (0.036,0.058) | 2.478 (1.927,3.131) | 2.186 (1.843,2.53) |
| Costa Rica | 0.034 (0.03,0.036) | 1.957 (1.763,2.119) | 0.179 (0.156,0.201) | 3.248 (2.844,3.643) | 1.988 (1.537,2.442) |
| El Salvador | 0.044 (0.032,0.069) | 1.48 (1.076,2.354) | 0.117 (0.071,0.16) | 1.794 (1.09,2.444) | 1.022 (0.839,1.205) |
| Honduras | 0.046 (0.03,0.071) | 2.218 (1.458,3.439) | 0.201 (0.101,0.298) | 3.414 (1.687,5.049) | 1.621 (1.448,1.794) |
| Nicaragua | 0.011 (0.009,0.017) | 0.744 (0.556,1.143) | 0.045 (0.027,0.062) | 0.978 (0.578,1.325) | 1.501 (1.204,1.799) |
| Panama | 0.016 (0.015,0.018) | 1.104 (0.998,1.203) | 0.128 (0.099,0.154) | 2.843 (2.205,3.439) | 3.784 (3.569,3.999) |

## Table S3 The DALYs number, age-standardized rates and temporal trends of Interstitial lung diseases in the Belt and Road Initiative countries from 1990 to 2021.

| **location** |  | | | | |
| --- | --- | --- | --- | --- | --- |
|  | **Number of cases, 1990  (thousands)** | **Age-standardised rate per 100,000 population, 1990** | **Number of cases, 2021  (thousands)** | **Age-standardised rate per 100,000 population, 2021** | **Estimated annual percentage change, 1990-2021** |
| Global | 1501.028 (1221.197,1850.557) | 37.148 (30.617,45.366) | 4042.15 (3489.795,4516.883) | 47.618 (41.258,53.165) | 0.952 (0.851,1.053) |
| Female | 647.895 (500.165,883.303) | 29.787 (23.229,40.191) | 1804.881 (1465.707,2216.376) | 39.486 (31.952,48.62) | 1.065 (0.957,1.173) |
| Male | 853.133 (666.667,1066.356) | 46.484 (36.62,57.591) | 2237.269 (1839.5,2555.2) | 57.788 (47.499,65.767) | 0.85 (0.755,0.946) |
| High SDI | 506.205 (470.841,546.44) | 46.538 (43.285,50.266) | 1500.93 (1354.746,1614.665) | 71.397 (65.27,76.565) | 1.537 (1.327,1.748) |
| High-middle SDI | 237.514 (213.56,266.885) | 23.853 (21.525,26.81) | 485.512 (430.896,541.669) | 25.479 (22.627,28.472) | 0.411 (0.318,0.505) |
| Middle SDI | 269.856 (212.977,366.219) | 25.076 (19.938,33.571) | 812.056 (695.675,984.262) | 30.999 (26.553,37.412) | 0.835 (0.755,0.914) |
| Low-middle SDI | 359.489 (213.852,552.929) | 57.351 (34.674,86.875) | 949.438 (662.365,1260.419) | 66.549 (46.338,88.364) | 0.637 (0.567,0.708) |
| Low SDI | 126.671 (60.835,188.619) | 53.162 (27.074,75.277) | 291.855 (178.64,406.44) | 56.021 (34.429,78.826) | 0.313 (0.203,0.423) |
| High-income North America | 210.856 (195.64,227.583) | 61.652 (57.124,66.567) | 582.575 (532.853,621.775) | 90.444 (83.276,96.544) | 1.279 (0.989,1.571) |
| Caribbean | 5.04 (3.866,6.914) | 18.07 (14.09,23.622) | 15.715 (12.779,19.981) | 29.998 (24.151,38.597) | 1.924 (1.732,2.116) |
| Andean Latin America | 33.945 (24.798,48.28) | 158.265 (116.076,221.595) | 121.673 (96.602,150.014) | 209.339 (165.807,257.665) | 1.466 (1.286,1.646) |
| Central Latin America | 33.623 (31.642,35.884) | 35.892 (33.808,38.252) | 158.077 (143.505,173.855) | 62.838 (57.108,69.096) | 1.918 (1.72,2.116) |
| Tropical Latin America | 27.651 (26.178,29.363) | 26.406 (24.856,28.109) | 102.784 (95.498,108.206) | 40.581 (37.645,42.74) | 1.33 (1.047,1.615) |
| North Africa and Middle East | 30.115 (21.398,44.298) | 16.194 (11.543,23.722) | 97.814 (75.696,134.363) | 20.109 (15.54,28.244) | 0.954 (0.809,1.1) |
| South Asia | 473.028 (268.377,740.983) | 81.676 (47.16,126.638) | 1312.644 (890.806,1740.639) | 89.796 (61.006,118.919) | 0.402 (0.332,0.472) |
| Central Sub-Saharan Africa | 8.097 (2.925,15.524) | 32.706 (12.315,68.731) | 20.545 (8.467,44.099) | 33.845 (13.174,79.52) | 0.085 (-0.019,0.189) |
| Eastern Sub-Saharan Africa | 22.391 (7.515,36.232) | 24.768 (9.296,43.148) | 47.826 (19.642,93.157) | 23.453 (9.359,46.538) | -0.289 (-0.341,-0.236) |
| Southern Sub-Saharan Africa | 13.659 (8.256,18.871) | 47.133 (27.177,67.434) | 26.228 (18.455,34.42) | 45.014 (31.731,59.127) | -0.317 (-0.563,-0.07) |
| Western Sub-Saharan Africa | 36.522 (13.623,55.767) | 38.641 (15.119,59.967) | 69.547 (29.751,115.085) | 31.333 (13.044,52.94) | -0.615 (-0.736,-0.494) |
| Oceania | 3.219 (2.153,4.773) | 69.387 (47.612,104.633) | 8.024 (5.581,12.403) | 74.63 (50.209,118.43) | 0.25 (0.169,0.331) |
| Central Asia | 20.131 (18.089,22.763) | 40.302 (35.894,45.693) | 19.79 (16.339,24.518) | 23.371 (19.372,28.781) | -1.984 (-2.396,-1.571) |
| Central Europe | 44.912 (41.926,48.293) | 30.888 (28.892,33.117) | 53.275 (48.499,58.218) | 26.9 (24.412,29.448) | -0.126 (-0.377,0.127) |
| East Asia | 115.445 (86.138,171.274) | 12.409 (9.415,18.31) | 234.266 (171.305,301.444) | 11.015 (8.056,14.138) | -0.123 (-0.281,0.034) |
| Eastern Europe | 79.279 (72.136,86.326) | 29.379 (26.781,31.975) | 34.252 (31.151,37.997) | 10.784 (9.778,11.996) | -4.556 (-5.262,-3.844) |
| High-income Asia Pacific | 132.227 (120.398,145.561) | 66.255 (60.195,72.902) | 432.703 (380.225,475.663) | 86.318 (76.637,94.927) | 0.705 (0.51,0.901) |
| Australasia | 5.708 (5.219,6.19) | 24.116 (22.114,26.101) | 33.036 (28.956,35.886) | 59.97 (53.126,64.898) | 3.096 (2.535,3.661) |
| Western Europe | 154.697 (143.923,165.739) | 27.507 (25.548,29.621) | 526.09 (478.286,559.276) | 56.372 (51.973,59.883) | 2.998 (2.649,3.348) |
| Southeast Asia | 22.495 (12.087,47.186) | 8.006 (4.354,16.313) | 59.048 (34.1,109.402) | 8.93 (5.184,16.515) | 0.372 (0.317,0.426) |
| Southern Latin America | 27.989 (26.106,29.94) | 60.448 (56.322,64.625) | 86.239 (79.828,92.166) | 98.578 (91.539,105.256) | 1.822 (1.514,2.131) |
| China | 112.642 (83.845,167.129) | 12.552 (9.491,18.567) | 222.288 (158.551,288.301) | 10.815 (7.701,13.972) | -0.23 (-0.384,-0.076) |
| Armenia | 1.713 (1.553,1.897) | 58.614 (53.592,64.519) | 1.27 (1.124,1.442) | 31.004 (27.467,35.137) | -0.9 (-1.874,0.083) |
| Azerbaijan | 1.894 (1.074,3.051) | 34.836 (19.04,58.099) | 2.36 (1.269,4.097) | 22.426 (12.209,37.198) | -1.757 (-2.109,-1.405) |
| Georgia | 1 (0.847,1.191) | 16.495 (13.996,19.63) | 0.85 (0.724,0.985) | 15.213 (12.984,17.64) | 1.214 (0.284,2.153) |
| Kazakhstan | 3.276 (2.909,3.655) | 24.351 (21.515,27.215) | 4.398 (3.454,5.532) | 23.596 (18.542,29.658) | -0.296 (-0.9,0.311) |
| Kyrgyzstan | 0.941 (0.818,1.037) | 28.405 (24.423,31.817) | 0.403 (0.335,0.472) | 7.348 (6.11,8.592) | -5.032 (-6.022,-4.032) |
| Mongolia | 0.885 (0.549,1.417) | 65.544 (42.383,98.44) | 0.946 (0.608,1.368) | 37.339 (24.018,54.785) | -2.278 (-2.496,-2.059) |
| Tajikistan | 2.568 (1.489,4.108) | 89.599 (51.58,144.854) | 3.854 (2.213,6.77) | 65.052 (37.994,114.845) | -1.319 (-1.451,-1.187) |
| Turkmenistan | 0.784 (0.671,0.885) | 34.663 (29.73,38.393) | 0.87 (0.696,1.118) | 18.2 (14.724,23.089) | -2.676 (-3.327,-2.02) |
| Uzbekistan | 7.071 (5.98,8.332) | 56.929 (46.998,69.243) | 4.839 (4.017,5.886) | 17.225 (14.589,20.743) | -4.55 (-5.273,-3.821) |
| Brunei Darussalam | 0.086 (0.061,0.116) | 79.545 (56.106,106.731) | 0.207 (0.152,0.268) | 60.897 (43.88,79.943) | -0.786 (-0.886,-0.686) |
| Republic of Korea | 13.334 (9.051,21.097) | 45.835 (30.248,74.134) | 46.534 (30.378,59.899) | 50.403 (33.11,64.868) | 0.797 (0.6,0.994) |
| Singapore | 0.533 (0.478,0.595) | 23.436 (21.274,25.857) | 2.058 (1.825,2.326) | 24.667 (21.843,27.748) | 0.556 (0.359,0.754) |
| Afghanistan | 0.246 (0.096,0.567) | 3.462 (1.351,7.814) | 0.985 (0.297,2.844) | 7.946 (2.171,21.999) | 3.516 (3.16,3.874) |
| Bahrain | 0.096 (0.067,0.159) | 50.47 (35.704,86.938) | 0.537 (0.331,0.741) | 62.066 (38.719,86.102) | 0.843 (0.553,1.133) |
| Iran (Islamic Republic of) | 0.71 (0.459,0.998) | 2.304 (1.494,3.182) | 2.571 (1.498,3.631) | 3.001 (1.74,4.261) | 1.188 (1.057,1.32) |
| Iraq | 1.562 (1.123,2.129) | 16.917 (11.899,23.641) | 5.106 (3.394,7.065) | 19.638 (13.046,26.709) | 0.433 (0.339,0.527) |
| Jordan | 0.916 (0.601,1.478) | 57.493 (38.258,93.38) | 4.099 (2.972,5.479) | 52.061 (37.655,70.17) | -0.266 (-0.443,-0.088) |
| Kuwait | 0.437 (0.397,0.474) | 60.478 (53.863,66.523) | 1.381 (1.161,1.621) | 45.685 (38.06,53.149) | 0.127 (-0.817,1.08) |
| Lebanon | 0.962 (0.479,1.754) | 43.479 (21.505,79.91) | 2.415 (1.824,3.44) | 39.314 (29.861,55.336) | 0.008 (-0.145,0.16) |
| Oman | 0.125 (0.084,0.198) | 14.838 (9.78,24.161) | 0.475 (0.291,0.718) | 20.144 (11.966,30.899) | 1.671 (1.332,2.011) |
| Palestine | 0.852 (0.588,1.173) | 90.526 (63.163,122.901) | 2.239 (1.58,2.892) | 87.139 (60.487,113.027) | 0.004 (-0.094,0.103) |
| Qatar | 0.036 (0.027,0.05) | 26.956 (20.169,41.271) | 0.259 (0.162,0.378) | 21.838 (13.478,32.236) | -0.447 (-0.658,-0.236) |
| Saudi Arabia | 6.174 (3.929,10.032) | 94.751 (62.059,156.57) | 26.638 (18.326,36.864) | 117.116 (78.333,162.695) | 1.04 (0.842,1.237) |
| Syrian Arab Republic | 1.028 (0.729,1.546) | 17.238 (11.827,26.448) | 3.023 (2.019,4.613) | 23.472 (15.826,35.966) | 1.14 (1.02,1.26) |
| Turkey | 9.157 (5.634,14.551) | 24.237 (14.919,38.363) | 25.363 (17.689,34.576) | 27.305 (19.22,37.295) | 0.77 (0.55,0.991) |
| United Arab Emirates | 0.807 (0.495,1.297) | 123.769 (76.774,198.239) | 4.382 (3.08,6.263) | 105.79 (75.904,151.273) | 1.044 (0.53,1.561) |
| Yemen | 0.199 (0.091,0.407) | 3.214 (1.456,6.169) | 1.178 (0.423,2.764) | 6.972 (2.327,16.872) | 3.173 (2.852,3.495) |
| Bangladesh | 37.318 (21.043,58.256) | 74.873 (42.553,117.449) | 88.725 (61.324,129.964) | 63.134 (43.437,92.233) | -0.637 (-0.758,-0.515) |
| Nepal | 10.138 (5.267,16.882) | 106.284 (56.488,175.621) | 27.664 (18.562,38.519) | 119.01 (79.897,165.7) | 0.507 (0.338,0.676) |
| Pakistan | 32.935 (20.838,47.379) | 57.907 (36.393,83.831) | 71.586 (48.23,97.472) | 58.583 (39.37,80.826) | -0.112 (-0.243,0.02) |
| Cambodia | 0.231 (0.069,0.68) | 4.538 (1.35,13.035) | 0.674 (0.249,1.644) | 5.27 (1.905,13.203) | 0.6 (0.533,0.667) |
| Indonesia | 7.739 (2.463,20.084) | 6.779 (2.145,17.42) | 20.229 (7.242,47.75) | 7.944 (2.791,19.059) | 0.577 (0.522,0.633) |
| Lao People's Democratic Republic | 0.164 (0.039,0.519) | 7.003 (1.735,20.922) | 0.368 (0.124,0.942) | 7.105 (2.328,18.174) | 0.017 (-0.006,0.039) |
| Malaysia | 2.055 (1.575,2.942) | 20.106 (15.227,28.71) | 6.816 (5.105,8.876) | 23.333 (17.304,30.581) | 0.572 (0.44,0.704) |
| Maldives | 0.154 (0.069,0.281) | 146.011 (75.039,253.358) | 0.329 (0.232,0.458) | 85.524 (62.557,112.497) | -1.891 (-1.973,-1.808) |
| Myanmar | 2.58 (0.613,8.523) | 10.443 (2.425,33.09) | 6.074 (2.005,15.214) | 12.5 (4.006,31.63) | 0.566 (0.512,0.62) |
| Philippines | 0.729 (0.558,0.934) | 2.164 (1.654,2.864) | 1.884 (1.393,2.382) | 2.075 (1.542,2.615) | -0.169 (-0.242,-0.095) |
| Sri Lanka | 3.275 (2.536,4.568) | 28.772 (22.276,40.321) | 6.422 (3.84,10.48) | 24.409 (14.575,39.234) | -0.508 (-0.707,-0.309) |
| Thailand | 2.634 (2.014,3.374) | 6.709 (5.117,8.912) | 5.829 (4.146,8.847) | 5.793 (4.123,8.709) | -0.769 (-0.89,-0.647) |
| Timor-Leste | 0.022 (0.006,0.058) | 5.909 (1.798,15.267) | 0.06 (0.02,0.141) | 6.433 (2.157,15.005) | 0.411 (0.217,0.606) |
| Viet Nam | 2.371 (0.791,5.757) | 5.625 (1.819,13.884) | 6.768 (2.511,15.187) | 6.889 (2.497,15.679) | 0.805 (0.738,0.871) |
| Albania | 0.662 (0.451,0.963) | 29.598 (19.934,43.332) | 0.882 (0.504,1.438) | 21.665 (12.408,35.787) | -1.008 (-1.162,-0.854) |
| Bosnia and Herzegovina | 0.976 (0.655,1.421) | 23.139 (15.303,33.826) | 0.884 (0.569,1.363) | 15.776 (10.363,24.027) | -1.404 (-1.512,-1.295) |
| Bulgaria | 1.521 (1.318,1.714) | 13.126 (11.515,14.755) | 1.713 (1.45,2.013) | 14.386 (12.179,16.94) | 0.313 (0.103,0.523) |
| Croatia | 0.553 (0.479,0.628) | 9.38 (8.169,10.63) | 0.793 (0.68,0.903) | 10.454 (8.98,12.004) | 0.778 (0.493,1.064) |
| Czechia | 2.839 (2.537,3.18) | 22.309 (20.057,24.795) | 7.628 (6.618,8.64) | 37.613 (32.57,42.633) | 2.597 (2.292,2.904) |
| Hungary | 3.905 (3.623,4.23) | 28.625 (26.495,31.005) | 5.917 (5.195,6.67) | 34.325 (30.032,38.712) | 1.077 (0.775,1.379) |
| North Macedonia | 0.143 (0.11,0.188) | 7.365 (5.664,9.702) | 0.236 (0.141,0.387) | 7.581 (4.514,12.241) | 0.168 (-0.008,0.345) |
| Montenegro | 0.033 (0.024,0.045) | 5.154 (3.679,7.073) | 0.042 (0.029,0.058) | 4.707 (3.345,6.459) | -0.212 (-0.257,-0.166) |
| Poland | 11.119 (10.325,12.001) | 25.746 (23.919,27.771) | 18.702 (17.067,20.554) | 28.95 (26.452,31.86) | 0.694 (0.306,1.084) |
| Romania | 19.157 (16.948,21.598) | 71.344 (63.512,79.398) | 10.894 (9.534,12.331) | 34.875 (30.692,39.52) | -2.14 (-2.471,-1.808) |
| Serbia | 1.613 (1.229,2.161) | 15.119 (11.438,20.646) | 1.93 (1.238,2.673) | 12.928 (8.357,17.776) | -0.327 (-0.423,-0.231) |
| Slovakia | 1.023 (0.776,1.442) | 17.444 (13.244,24.484) | 1.524 (0.95,2.233) | 17.348 (10.891,25.456) | 0.302 (0.174,0.43) |
| Slovenia | 0.649 (0.595,0.708) | 27.363 (25.124,29.895) | 1.355 (1.154,1.559) | 32.509 (27.858,37.513) | 0.769 (0.518,1.02) |
| Belarus | 5.149 (4.38,5.845) | 41.501 (35.293,47.524) | 1.287 (1.062,1.527) | 9.01 (7.421,10.691) | -5.854 (-6.272,-5.435) |
| Estonia | 1.204 (1.056,1.38) | 59.714 (52.287,68.239) | 0.222 (0.189,0.264) | 9.087 (7.516,10.935) | -7.129 (-8.874,-5.35) |
| Latvia | 2.977 (2.649,3.316) | 83.998 (74.702,93.182) | 0.329 (0.28,0.384) | 9.373 (7.863,11.124) | -7.79 (-9.395,-6.157) |
| Lithuania | 1.495 (1.289,1.769) | 33.606 (29.133,39.615) | 0.28 (0.235,0.329) | 5.631 (4.656,6.782) | -6.464 (-8.102,-4.796) |
| Republic of Moldova | 0.63 (0.554,0.721) | 14.637 (12.917,16.635) | 0.162 (0.119,0.212) | 3.061 (2.221,4.045) | -5.642 (-6.923,-4.343) |
| Russian Federation | 41.661 (35.36,47.064) | 23.731 (20.172,26.805) | 25.018 (22.853,27.525) | 11.335 (10.339,12.501) | -3.817 (-4.777,-2.848) |
| Ukraine | 26.163 (23.335,28.938) | 38.636 (34.604,42.815) | 6.954 (5.329,8.778) | 10.47 (8.069,13.205) | -5.436 (-5.886,-4.983) |
| Austria | 1.764 (1.553,1.977) | 15.555 (13.574,17.665) | 4.781 (4.288,5.267) | 27.319 (24.714,30.168) | 2.644 (2.361,2.928) |
| Cyprus | 0.933 (0.625,1.41) | 131.367 (90.083,197.626) | 1.666 (1.199,2.196) | 81.337 (59.675,106.6) | -1.397 (-1.595,-1.198) |
| Greece | 1.532 (1.405,1.681) | 10.73 (9.867,11.72) | 9.475 (8.535,10.295) | 41.76 (38.481,44.905) | 5.301 (4.643,5.964) |
| Italy | 7.776 (6.451,9.199) | 9.678 (7.971,11.497) | 59.745 (53.515,64.591) | 43.505 (39.946,46.84) | 5.973 (4.833,7.125) |
| Luxembourg | 0.095 (0.087,0.105) | 17.639 (15.968,19.372) | 0.392 (0.346,0.438) | 37.434 (33.203,41.863) | 3.091 (2.634,3.549) |
| Malta | 0.168 (0.153,0.182) | 39.564 (36.147,42.863) | 0.861 (0.759,0.963) | 88.885 (79.163,99.476) | 2.854 (2.401,3.31) |
| Portugal | 2.613 (2.408,2.822) | 19.66 (18.146,21.197) | 12.588 (11.193,13.719) | 52.276 (47.328,56.631) | 3.817 (3.216,4.422) |
| Algeria | 0.489 (0.242,0.824) | 3.44 (1.696,5.865) | 2.776 (1.103,6.568) | 7.417 (2.816,18.153) | 3.097 (2.839,3.356) |
| Egypt | 5.124 (3.772,7.073) | 17.23 (13.037,23.962) | 8.174 (6.084,10.662) | 12.418 (9.389,16.423) | -1.414 (-1.69,-1.136) |
| Libya | 0.084 (0.042,0.152) | 3.782 (1.88,6.842) | 0.576 (0.174,1.654) | 9.744 (2.778,28.88) | 3.827 (3.53,4.125) |
| Morocco | 0.521 (0.253,0.907) | 3.202 (1.555,5.43) | 2.705 (1.007,5.895) | 7.649 (2.759,17.006) | 3.469 (3.198,3.74) |
| Tunisia | 0.19 (0.096,0.315) | 3.386 (1.701,5.706) | 0.963 (0.384,2.476) | 7.212 (2.827,18.622) | 2.934 (2.723,3.146) |
| Angola | 1.328 (0.442,2.394) | 29.073 (10.785,56.233) | 3.369 (1.415,5.841) | 24.935 (10.372,45.618) | -0.632 (-0.682,-0.581) |
| Central African Republic | 0.438 (0.134,0.858) | 35.456 (12.244,67.981) | 0.842 (0.301,1.631) | 33.741 (12.825,69.872) | -0.22 (-0.288,-0.152) |
| Congo | 0.432 (0.141,0.855) | 37.647 (12.968,78.709) | 0.95 (0.398,1.684) | 31.684 (13.249,58.934) | -0.722 (-0.812,-0.631) |
| Democratic Republic of the Congo | 5.632 (2.051,11.528) | 33.018 (12.289,73.75) | 14.92 (5.882,35.891) | 37.123 (14.129,97.53) | 0.399 (0.263,0.535) |
| Equatorial Guinea | 0.07 (0.022,0.139) | 33.32 (11.513,62.627) | 0.168 (0.065,0.342) | 27.553 (11.026,56.548) | -0.704 (-0.77,-0.638) |
| Gabon | 0.196 (0.071,0.348) | 33.496 (12.435,60.222) | 0.295 (0.119,0.608) | 26.816 (10.862,55.941) | -0.826 (-0.887,-0.764) |
| Burundi | 0.873 (0.248,1.563) | 30.301 (10.033,53.057) | 1.426 (0.561,2.817) | 24.481 (9.196,50.039) | -0.901 (-1.048,-0.753) |
| Comoros | 0.065 (0.021,0.121) | 27.899 (9.619,52.832) | 0.116 (0.049,0.236) | 22.005 (9.15,45.413) | -0.96 (-1.199,-0.721) |
| Djibouti | 0.039 (0.013,0.07) | 22.854 (8.603,40.111) | 0.133 (0.054,0.272) | 18.399 (7.417,36.503) | -0.802 (-0.917,-0.686) |
| Eritrea | 0.438 (0.113,0.865) | 29.04 (9.058,52.148) | 0.863 (0.343,1.448) | 25.353 (10.482,42.285) | -0.528 (-0.616,-0.44) |
| Ethiopia | 5.419 (1.473,9.657) | 22.801 (6.902,39.473) | 9.412 (3.683,18.421) | 18.247 (6.706,37.185) | -0.992 (-1.097,-0.886) |
| Kenya | 2.208 (0.756,5.543) | 22.836 (7.427,61.199) | 7.13 (2.397,20.417) | 28.108 (8.929,84.458) | 0.785 (0.694,0.877) |
| Madagascar | 2.43 (0.824,4.01) | 38.57 (14.903,67.365) | 5.846 (2.357,11.428) | 40.756 (16.485,80.385) | 0.075 (0.027,0.124) |
| Malawi | 0.999 (0.318,1.7) | 21.196 (7.589,37.98) | 2.145 (0.879,4.025) | 24.081 (9.771,47.046) | 0.29 (0.198,0.382) |
| Mozambique | 1.331 (0.403,2.436) | 17.719 (6.386,31.733) | 2.774 (1.098,5.3) | 19.8 (8.238,37.673) | 0.499 (0.409,0.59) |
| Rwanda | 1.164 (0.331,2.122) | 32.309 (10.512,55.846) | 1.794 (0.724,3.869) | 24.339 (9.472,53.632) | -1.435 (-1.675,-1.194) |
| Seychelles | 0.003 (0.001,0.008) | 5.978 (2.162,14.395) | 0.006 (0.002,0.015) | 5.446 (2.062,12.711) | -0.22 (-0.292,-0.148) |
| Somalia | 0.914 (0.239,1.766) | 29.781 (9.069,54.941) | 1.963 (0.703,3.663) | 24.728 (9.887,44.633) | -0.617 (-0.686,-0.547) |
| United Republic of Tanzania | 2.646 (0.956,4.53) | 20.037 (7.641,36.35) | 6.019 (2.396,12.685) | 19.595 (7.763,41.927) | -0.15 (-0.182,-0.118) |
| Uganda | 2.14 (0.749,4.082) | 27.6 (9.875,54.598) | 4.674 (1.797,10.621) | 25.519 (9.763,58.165) | -0.53 (-0.625,-0.434) |
| Zambia | 0.824 (0.281,1.454) | 23.135 (8.873,38.803) | 2.287 (0.943,4.4) | 26.189 (10.883,50.651) | 0.376 (0.296,0.456) |
| Botswana | 0.267 (0.081,0.542) | 46.42 (13.808,94.275) | 0.489 (0.169,0.955) | 32.774 (11.232,64.798) | -1.082 (-1.219,-0.944) |
| Lesotho | 0.305 (0.092,0.587) | 35.401 (10.428,69.118) | 0.418 (0.129,0.829) | 37.043 (11.27,73.767) | 0.411 (0.226,0.597) |
| Namibia | 0.301 (0.089,0.598) | 45.592 (13.236,92.511) | 0.599 (0.189,1.215) | 42.238 (13.161,85.494) | -0.365 (-0.464,-0.266) |
| South Africa | 12.121 (7.743,16.435) | 54.313 (33.062,76.235) | 23.448 (17.434,32.908) | 50.17 (37.168,70.117) | -0.479 (-0.739,-0.218) |
| Zimbabwe | 0.52 (0.183,0.862) | 12.438 (4.101,21.338) | 1.018 (0.344,1.864) | 13.765 (4.355,25.669) | 0.372 (0.22,0.525) |
| Benin | 1.008 (0.374,1.654) | 45.235 (17.409,76.933) | 1.888 (0.743,3.704) | 31.16 (12.1,65.222) | -1.143 (-1.409,-0.876) |
| Burkina Faso | 1.371 (0.432,2.323) | 28.183 (9.759,46.487) | 2.43 (0.945,4.14) | 21.843 (8.891,40.013) | -0.857 (-0.997,-0.717) |
| Cameroon | 2.588 (0.988,4.219) | 53.024 (20.886,89.532) | 5.502 (2.217,9.768) | 36.766 (14.626,67.231) | -1.098 (-1.325,-0.871) |
| Cabo Verde | 0.138 (0.042,0.279) | 58.187 (17.606,118.158) | 0.116 (0.048,0.222) | 25.03 (10.335,47.291) | -2.431 (-3.116,-1.742) |
| Chad | 1.259 (0.439,2.179) | 41.295 (14.79,72.24) | 2.411 (0.992,4.285) | 35.386 (14.635,65.227) | -0.359 (-0.544,-0.173) |
| Côte d'Ivoire | 2.206 (0.793,3.732) | 48.113 (18.06,83.197) | 4.416 (1.664,8.19) | 33.593 (12.55,64.164) | -1.061 (-1.299,-0.822) |
| Gambia | 0.192 (0.067,0.329) | 47.674 (17.258,84.537) | 0.438 (0.185,0.848) | 38.461 (15.792,76.359) | -0.694 (-1.006,-0.382) |
| Ghana | 2.386 (0.878,3.94) | 33.509 (12.929,53.641) | 6.346 (2.8,10.447) | 33.886 (14.893,55.114) | 0.201 (0.103,0.299) |
| Guinea | 1.502 (0.564,2.454) | 42.698 (16.727,70.611) | 2.208 (0.92,4.24) | 34.756 (14.277,70.173) | -0.494 (-0.694,-0.295) |
| Guinea-Bissau | 0.26 (0.076,0.512) | 58.023 (18.358,108.358) | 0.362 (0.144,0.613) | 40.531 (16.88,69.972) | -0.999 (-1.244,-0.754) |
| Liberia | 0.565 (0.198,0.987) | 44.81 (16.3,82.221) | 0.894 (0.339,1.811) | 35.063 (12.816,73.935) | -0.668 (-0.958,-0.377) |
| Mali | 2.838 (0.909,4.78) | 65.424 (22.077,109.515) | 5.892 (2.197,11.747) | 57.329 (20.496,118.3) | -0.371 (-0.468,-0.274) |
| Mauritania | 0.492 (0.183,0.809) | 46.114 (17.219,76.217) | 0.685 (0.293,1.255) | 29.51 (12.316,54.899) | -1.457 (-1.81,-1.104) |
| Niger | 1.476 (0.479,2.668) | 46.118 (16.699,81.235) | 3.03 (1.24,5.699) | 32.311 (12.63,63.882) | -0.962 (-1.234,-0.689) |
| Nigeria | 14.95 (5.849,23.658) | 32.722 (12.893,52.286) | 26.938 (10.935,48.502) | 27.077 (10.915,49.967) | -0.636 (-0.696,-0.576) |
| Sao Tome and Principe | 0.065 (0.023,0.115) | 95.877 (34.102,172.7) | 0.108 (0.041,0.24) | 87.608 (34.053,190.096) | -0.356 (-0.449,-0.264) |
| Senegal | 1.646 (0.566,2.679) | 45.84 (16.683,75.781) | 2.917 (1.232,5.465) | 34.253 (14.069,65.652) | -0.738 (-1.106,-0.368) |
| Sierra Leone | 0.922 (0.313,1.566) | 41.604 (14.568,72.912) | 1.378 (0.528,2.661) | 31.574 (12.04,63.857) | -0.712 (-0.99,-0.433) |
| Togo | 0.657 (0.239,1.093) | 45.14 (17.441,78.805) | 1.586 (0.624,3.083) | 36.253 (13.882,73.85) | -0.614 (-0.845,-0.382) |
| South Sudan | 0.888 (0.282,1.761) | 29.318 (10.239,57.562) | 1.201 (0.467,2.321) | 25.972 (10.476,53.425) | -0.519 (-0.752,-0.286) |
| Sudan | 0.383 (0.161,0.84) | 3.409 (1.46,6.97) | 1.88 (0.619,4.734) | 7.821 (2.46,19.965) | 3.365 (3.073,3.658) |
| Guyana | 0.146 (0.129,0.163) | 34.5 (30.328,38.763) | 0.321 (0.241,0.413) | 47.164 (35.883,60.387) | 2.202 (1.415,2.994) |
| Suriname | 0.073 (0.058,0.104) | 26.513 (20.798,38.54) | 0.216 (0.134,0.299) | 34.675 (21.389,47.918) | 1.358 (1.164,1.552) |
| Bolivia (Plurinational State of) | 5.439 (2.679,10.909) | 165.254 (83.416,319.274) | 15.874 (10.498,22.568) | 184.192 (121.938,261.468) | 0.538 (0.46,0.616) |
| Ecuador | 3.375 (3.073,3.726) | 62.343 (56.336,69.213) | 23.487 (19.334,28.547) | 147.186 (121.707,177.957) | 3.904 (3.418,4.392) |
| Peru | 25.131 (18.466,34.915) | 198.954 (147.16,272.019) | 82.312 (59.629,106.113) | 246.21 (178.271,317.786) | 1.204 (0.977,1.432) |
| Venezuela (Bolivarian Republic of) | 2.467 (2.264,2.693) | 22.291 (20.366,24.417) | 10.58 (8.172,13.348) | 36.207 (28.124,45.318) | 1.963 (1.704,2.224) |
| Brazil | 27.15 (25.67,28.867) | 26.538 (24.962,28.306) | 100.994 (93.922,106.357) | 40.844 (37.915,43.04) | 1.327 (1.039,1.616) |
| Argentina | 18.269 (16.721,19.843) | 56.816 (52.007,61.727) | 41.402 (38.08,45.001) | 73.842 (67.964,80.078) | 1.132 (0.784,1.481) |
| Chile | 8.498 (7.975,8.998) | 84.291 (79.012,89.344) | 41.509 (38.124,44.276) | 161.708 (148.71,172.348) | 2.289 (1.964,2.616) |
| Uruguay | 1.22 (1.119,1.327) | 31.876 (29.297,34.685) | 3.323 (3.052,3.575) | 59.551 (55.204,63.822) | 2.317 (2.074,2.56) |
| Fiji | 0.138 (0.103,0.185) | 23.426 (17.457,31.046) | 0.208 (0.131,0.288) | 24.128 (15.123,33.141) | 0.215 (0.101,0.33) |
| Kiribati | 0.039 (0.018,0.069) | 63.921 (30.898,109.568) | 0.067 (0.036,0.113) | 63.298 (34.592,106.976) | -0.111 (-0.16,-0.061) |
| Micronesia (Federated States of) | 0.06 (0.031,0.097) | 73.583 (40.402,121.252) | 0.054 (0.033,0.093) | 58.782 (35.484,101.822) | -0.717 (-0.797,-0.637) |
| Papua New Guinea | 2.267 (1.372,3.464) | 80.094 (50.853,125.451) | 6.598 (4.438,10.357) | 86.942 (55.917,141.026) | 0.282 (0.203,0.362) |
| Samoa | 0.087 (0.055,0.14) | 66.632 (42.292,108.663) | 0.104 (0.064,0.177) | 58.854 (36.488,100.234) | -0.318 (-0.41,-0.226) |
| Solomon Islands | 0.069 (0.045,0.102) | 32.643 (21.694,48.606) | 0.173 (0.114,0.296) | 34.816 (22.944,59.077) | 0.25 (0.144,0.355) |
| Tonga | 0.032 (0.022,0.049) | 42.608 (29.579,67.063) | 0.039 (0.024,0.067) | 42.592 (25.748,73.561) | 0.133 (0.064,0.202) |
| Vanuatu | 0.073 (0.036,0.115) | 65.124 (34.691,105.02) | 0.165 (0.088,0.283) | 64.066 (34.784,111.264) | -0.23 (-0.329,-0.131) |
| Cook Islands | 0.007 (0.005,0.011) | 44.852 (31.56,71.653) | 0.007 (0.004,0.012) | 31.239 (17.274,56.502) | -1.366 (-1.495,-1.236) |
| Nauru | 0.006 (0.003,0.011) | 73.735 (41.224,131.878) | 0.006 (0.003,0.012) | 67.924 (35.279,125.921) | -0.299 (-0.34,-0.259) |
| Niue | 0.001 (0.001,0.002) | 63.96 (45.06,96.032) | 0.001 (0.001,0.002) | 85.006 (56.186,133.466) | -0.297 (-0.749,0.157) |
| New Zealand | 1.125 (1.034,1.228) | 28.372 (26.13,30.876) | 4.827 (4.349,5.266) | 56.319 (51.147,60.992) | 2.246 (2.036,2.457) |
| Antigua and Barbuda | 0.006 (0.005,0.006) | 10.071 (9.084,11.258) | 0.023 (0.022,0.025) | 23.038 (21.529,24.888) | 3.276 (2.955,3.598) |
| Barbados | 0.058 (0.053,0.063) | 20.101 (18.176,22.004) | 0.196 (0.159,0.235) | 40.217 (32.679,48.47) | 3.029 (2.704,3.356) |
| Cuba | 0.578 (0.513,0.648) | 5.543 (4.929,6.204) | 1.608 (1.386,1.821) | 9.053 (7.822,10.283) | 1.959 (1.703,2.217) |
| Dominica | 0.011 (0.008,0.016) | 17.699 (13.448,26.372) | 0.022 (0.014,0.03) | 27.227 (18.045,37.715) | 1.591 (1.458,1.724) |
| Dominican Republic | 0.391 (0.255,0.677) | 8.765 (5.608,16.119) | 1.371 (0.813,2.305) | 13.218 (7.807,22.066) | 1.683 (1.513,1.853) |
| Grenada | 0.013 (0.011,0.015) | 17.59 (15.063,20.634) | 0.043 (0.037,0.049) | 38.807 (33.464,44.033) | 3.094 (2.767,3.423) |
| Jamaica | 0.193 (0.175,0.213) | 10.371 (9.398,11.446) | 0.779 (0.602,0.981) | 25.287 (19.468,31.853) | 3.431 (3.058,3.806) |
| Trinidad and Tobago | 0.318 (0.293,0.345) | 36.303 (33.369,39.4) | 1.118 (0.884,1.412) | 61.047 (48.376,76.786) | 2.334 (2.045,2.624) |
| Costa Rica | 0.918 (0.848,0.99) | 48.219 (44.262,52.147) | 4.208 (3.768,4.663) | 77.17 (69.18,85.369) | 1.728 (1.424,2.033) |
| El Salvador | 1.242 (0.971,1.812) | 37.402 (29.14,54.067) | 2.829 (1.89,3.718) | 45.216 (30.25,59.362) | 0.973 (0.802,1.145) |
| Honduras | 1.593 (1.05,2.504) | 59.671 (40.436,90.75) | 5.758 (3.206,8.488) | 83.782 (45.773,121.531) | 1.266 (1.116,1.415) |
| Nicaragua | 0.376 (0.306,0.52) | 19.564 (15.642,28.075) | 1.32 (0.875,1.709) | 25.539 (16.812,33.275) | 1.452 (1.201,1.704) |
| Panama | 0.477 (0.439,0.517) | 28.894 (26.532,31.404) | 2.798 (2.219,3.319) | 63.218 (50.111,75.038) | 3.173 (2.977,3.369) |

## Table S4 The age-standardized rates and temporal trends of Interstitial lung diseases by age-groups in China from 1990 to 2021

| **age** | **ASIR(per 100,000)** | | | **ASMR(per 100,000)** | | | **ASDR(per 100,000)** | | |
| --- | --- | --- | --- | --- | --- | --- | --- | --- | --- |
|  | **1990** | **2021** | **EAPC,1990-2021** | **1990** | **2021** | **EAPC,1990-2021** | **1990** | **2021** | **EAPC,1990-2021** |
| <5 years | / | / | / | 0.104(0.042,0.204) | 0.007(0.003,0.012) | -8.391(-8.992,-7.787) | 8.992(3.659,17.7) | 0.574(0.26,1.038) | -8.395(-8.997,-7.789) |
| 5-9 years | / | / | / | 0.050(0.03,0.08) | 0.009(0.005,0.015) | -5.766(-6.178,-5.351) | 4.150(2.523,6.631) | 0.744(0.399,1.252) | -5.765(-6.179,-5.349) |
| 10-14 years | / | / | / | 0.015(0.009,0.024) | 0.004(0.002,0.007) | -4.355(-4.83,-3.879) | 1.169(0.729,1.85) | 0.320(0.176,0.52) | -4.358(-4.835,-3.878) |
| 15-19 years | 0.236(0.058,0.522) | 0.140(0.032,0.325) | 2.776(2.371,3.183) | 0.016(0.01,0.027) | 0.009(0.006,0.015) | -1.759(-1.853,-1.666) | 1.172(0.777,1.995) | 0.707(0.445,1.094) | -1.783(-1.865,-1.7) |
| 20-24 years | 0.712(0.391,1.163) | 0.383(0.205,0.633) | 2.127(1.862,2.394) | 0.018(0.012,0.029) | 0.015(0.009,0.022) | -0.491(-0.684,-0.298) | 1.492(1.064,2.313) | 1.192(0.782,1.71) | -0.861(-0.98,-0.741) |
| 25-29 years | 1.073(0.521,1.748) | 0.571(0.273,0.989) | 1.67(1.405,1.937) | 0.026(0.018,0.043) | 0.024(0.016,0.035) | -0.022(-0.312,0.269) | 2.437(1.774,3.547) | 1.908(1.36,2.67) | -0.802(-0.898,-0.706) |
| 30-34 years | 1.329(0.624,2.239) | 0.827(0.381,1.369) | 1.287(1.025,1.549) | 0.039(0.026,0.062) | 0.032(0.019,0.05) | -0.247(-0.501,0.007) | 3.618(2.621,5.229) | 2.631(1.816,3.709) | -1.094(-1.193,-0.995) |
| 35-39 years | 1.731(0.897,2.974) | 1.321(0.697,2.245) | -2.321(-2.727,-1.914) | 0.062(0.042,0.101) | 0.051(0.031,0.076) | -0.711(-0.836,-0.586) | 5.346(3.872,7.713) | 4.000(2.746,5.418) | -1.252(-1.39,-1.114) |
| 40-44 years | 2.431(1.203,3.909) | 2.139(1.118,3.352) | -2.87(-3.411,-2.326) | 0.098(0.066,0.172) | 0.081(0.046,0.118) | -0.576(-0.684,-0.467) | 7.734(5.505,11.829) | 6.022(4.095,8.355) | -0.969(-1.062,-0.876) |
| 45-49 years | 3.621(1.986,5.933) | 3.516(2.015,5.428) | -2.897(-3.428,-2.364) | 0.148(0.101,0.248) | 0.119(0.066,0.178) | -0.341(-0.548,-0.135) | 10.746(7.858,15.916) | 8.582(6.019,12.106) | -0.595(-0.709,-0.48) |
| 50-54 years | 4.704(2.459,7.223) | 5.060(2.81,7.416) | -2.076(-2.417,-1.733) | 0.272(0.184,0.495) | 0.209(0.123,0.32) | -0.6(-0.753,-0.446) | 16.575(11.571,25.403) | 13.431(9.63,18.458) | -0.506(-0.628,-0.384) |
| 55-59 years | 5.592(3.256,8.418) | 6.901(4.29,9.995) | -1.109(-1.265,-0.952) | 0.550(0.362,1) | 0.435(0.239,0.644) | -0.609(-0.737,-0.482) | 26.472(18.904,41.858) | 22.428(15.831,30.813) | -0.347(-0.476,-0.219) |
| 60-64 years | 6.114(3.506,9.468) | 9.037(5.585,13.084) | -0.426(-0.513,-0.339) | 0.913(0.626,1.586) | 0.794(0.459,1.145) | -0.205(-0.344,-0.066) | 36.512(26.482,57.13) | 34.021(23.832,46.048) | 0.071(-0.103,0.246) |
| 65-69 years | 5.669(3.321,8.879) | 9.408(5.957,13.933) | 0.142(-0.025,0.309) | 1.504(1.074,2.449) | 1.330(0.797,1.915) | -0.149(-0.296,-0.003) | 48.385(36.012,69.952) | 46.650(33.843,62.71) | 0.217(0.029,0.406) |
| 70-74 years | 5.050(2.893,7.671) | 9.395(5.465,13.474) | 0.752(0.449,1.056) | 2.592(1.882,4.54) | 2.401(1.456,3.45) | 0.069(-0.113,0.251) | 64.443(49.724,103.958) | 64.982(45.044,86.784) | 0.411(0.196,0.626) |
| 75-79 years | 4.640(2.704,7.652) | 9.222(5.71,13.815) | 1.493(1.062,1.926) | 4.175(3.072,6.992) | 4.192(2.388,6.051) | 0.445(0.234,0.657) | 79.632(61.294,127.254) | 85.405(57.958,115.413) | 0.722(0.471,0.974) |
| 80-84 years | 5.695(3.528,8.785) | 10.497(6.825,16.105) | 2.141(1.633,2.651) | 4.812(3.582,8.193) | 5.447(3.055,7.777) | 0.985(0.717,1.254) | 72.030(55.045,114.293) | 86.529(55.717,114.891) | 1.245(0.931,1.561) |
| 85-89 years | 7.809(5.32,10.757) | 12.895(9.197,17.271) | 2.609(2.059,3.163) | 6.704(4.789,11.564) | 8.693(4.573,12.164) | 1.5(1.22,1.782) | 76.740(57.073,125.091) | 103.007(63.391,137.232) | 1.659(1.363,1.956) |
| 90-94 years | 9.522(5.954,14.12) | 14.254(9.214,20.445) | 3.045(2.474,3.619) | 9.907(7.361,16.217) | 11.166(6.22,14.996) | 0.982(0.707,1.257) | 93.937(71.842,150.216) | 110.343(66.418,144.353) | 1.135(0.848,1.424) |
| 95+ years | 10.579(5.954,16.077) | 14.839(8.371,22.233) | 3.321(2.759,3.887) | 11.610(8.208,17.704) | 13.901(7.646,19.704) | 0.977(0.699,1.256) | 102.507(73.947,155.215) | 124.707(73.69,172.508) | 1.046(0.759,1.335) |

## Table S5 Joinpoint regression analysis for global and China trends of ASIR, ASMR, and ASDR (per 100,000) of ILD from 1990 to 2021

| ASIR | | | |
| --- | --- | --- | --- |
| location | Segment(year) | APC(%)(95% CI) | P-Value |
| Global | 1990-1995 | 0.747(0.66,0.811) | <0001 |
|  | 1995-1999 | 1.348(1.237,1.482) | <0001 |
|  | 1999-2006 | 0.82(0.777,0.857) | <0001 |
|  | 2006-2009 | 1.8(1.707,1.891) | <0001 |
|  | 2009-2012 | 0.289(0.149,0.375) | <0001 |
|  | 2012-2021 | -0.248(-0.278,-0.225) | <0001 |
|  | AAPC(%s) | 0.607(0.599,0.615) | <0001 |
| China | 1990-2000 | -0.192(-0.432,-0.05) | 0.011 |
|  | 2000-2005 | 0.733(0.387,1.474) | <0001 |
|  | 2005-2010 | 5.936(5.662,6.246) | 0.002 |
|  | 2010-2021 | -1.138(-1.256,-1.025) | <0001 |
|  | AAPC(%s) | 0.581(0.539,0.618) | <0001 |
|  |  |  |  |
| ASMR | | | |
| location | Segment(year) | APC(%) | P-Value |
| Global | 1990-1993 | 1.353(0.523,1.968) | 0.001 |
|  | 1993-2004 | 2.284(2.181,2.582) | <0001 |
|  | 2004-2014 | 1.37(1.238,1.583) | <0001 |
|  | 2014-2019 | 0.354(-0.01,0.86) | 0.053 |
|  | 2019-2021 | -1.781(-2.502,-0.792) | <0001 |
|  | AAPC(%s) | 1.32(1.273,1.371) | <0001 |
| China | 1990-1999 | -0.895(-1.077,-0.752) | 0.002 |
|  | 1999-2006 | 0.666(0.295,0.926) | 0.028 |
|  | 2006-2010 | 3.167(2.413,3.79) | 0.001 |
|  | 2010-2013 | -0.61(-1.118,1.007) | 0.117 |
|  | 2013-2021 | -1.554(-1.83,-1.425) | <0001 |
|  | AAPC(%s) | -0.172(-0.21,-0.141) | <0001 |
|  |  |  |  |
| ASDR | | | |
| location | Segment(year) | APC(%) | P-Value |
| Global | 1990-2004 | 1.372(1.313,1.462) | <0001 |
|  | 2004-2012 | 0.95(0.768,1.144) | <0001 |
|  | 2012-2019 | 0.232(0.049,0.44) | 0.021 |
|  | 2019-2021 | -1.342(-1.867,-0.553) | <0001 |
|  | AAPC(%s) | 0.828(0.797,0.862) | <0001 |
| China | 1990-2001 | -1.015(-1.148,-0.918) | 0.003 |
|  | 2001-2006 | -0.018(-0.932,0.363) | 0.788 |
|  | 2006-2009 | 3.164(0.034,3.521) | 0.043 |
|  | 2009-2012 | -0.036(-0.532,3.124) | 0.911 |
|  | 2012-2018 | -1.764(-2.224,-0.211) | 0.008 |
|  | 2018-2021 | -1.01(-1.582,-0.401) | 0.003 |
|  | AAPC(%s) | -0.509(-0.549,-0.483) | <0001 |

## Table S6 Predicting the burden of ASDR of ILD in global and China.

| location | year | DALYs cases | year | DALYs cases | year | DALYs cases |
| --- | --- | --- | --- | --- | --- | --- |
|  | Both | | Male | | Female | |
| Global | 2022 | 47.71(47.07,48.36) | 2022 | 58.07(57.24,58.9) | 2022 | 39.53(38.97,40.08) |
|  | 2023 | 47.29(46.03,48.55) | 2023 | 57.86(56.44,59.27) | 2023 | 39.49(38.42,40.56) |
|  | 2024 | 46.78(45.05,48.52) | 2024 | 57.29(55.55,59.04) | 2024 | 39.46(37.94,40.98) |
|  | 2025 | 46.62(44.31,48.93) | 2025 | 57.06(54.91,59.21) | 2025 | 39.42(37.46,41.39) |
|  | 2026 | 46.41(43.35,49.46) | 2026 | 57(54.26,59.75) | 2026 | 39.38(36.97,41.8) |
|  | 2027 | 45.99(42.17,49.81) | 2027 | 56.73(53.38,60.08) | 2027 | 39.35(36.46,42.24) |
|  | 2028 | 45.67(41.08,50.26) | 2028 | 56.37(52.45,60.29) | 2028 | 39.31(35.94,42.69) |
|  | 2029 | 45.45(40,50.89) | 2029 | 56.16(51.61,60.7) | 2029 | 39.28(35.4,43.15) |
|  | 2030 | 45.13(38.77,51.5) | 2030 | 55.97(50.74,61.21) | 2030 | 39.24(34.85,43.64) |
|  | 2031 | 44.79(37.49,52.09) | 2031 | 55.7(49.75,61.66) | 2031 | 39.2(34.27,44.14) |
|  | Both | | Male | | Female | |
| China | 2022 | 10.86(10.68,11.04) | 2022 | 13.98(13.7,14.26) | 2022 | 8.34(8.2,8.48) |
|  | 2023 | 10.98(10.62,11.34) | 2023 | 14.04(13.48,14.61) | 2023 | 8.34(8.05,8.63) |
|  | 2024 | 11.16(10.61,11.7) | 2024 | 14.16(13.33,14.99) | 2024 | 8.34(7.89,8.79) |
|  | 2025 | 11.37(10.67,12.07) | 2025 | 14.32(13.27,15.37) | 2025 | 8.34(7.72,8.96) |
|  | 2026 | 11.59(10.76,12.42) | 2026 | 14.49(13.27,15.72) | 2026 | 8.34(7.56,9.12) |
|  | 2027 | 11.79(10.88,12.71) | 2027 | 14.66(13.32,16) | 2027 | 8.34(7.4,9.28) |
|  | 2028 | 11.97(11.01,12.93) | 2028 | 14.81(13.4,16.22) | 2028 | 8.34(7.24,9.44) |
|  | 2029 | 12.1(11.11,13.08) | 2029 | 14.93(13.48,16.38) | 2029 | 8.34(7.09,9.59) |
|  | 2030 | 12.17(11.18,13.16) | 2030 | 15.02(13.56,16.48) | 2030 | 8.34(6.94,9.74) |
|  | 2031 | 12.2(11.21,13.19) | 2031 | 15.07(13.61,16.53) | 2031 | 8.34(6.8,9.88) |


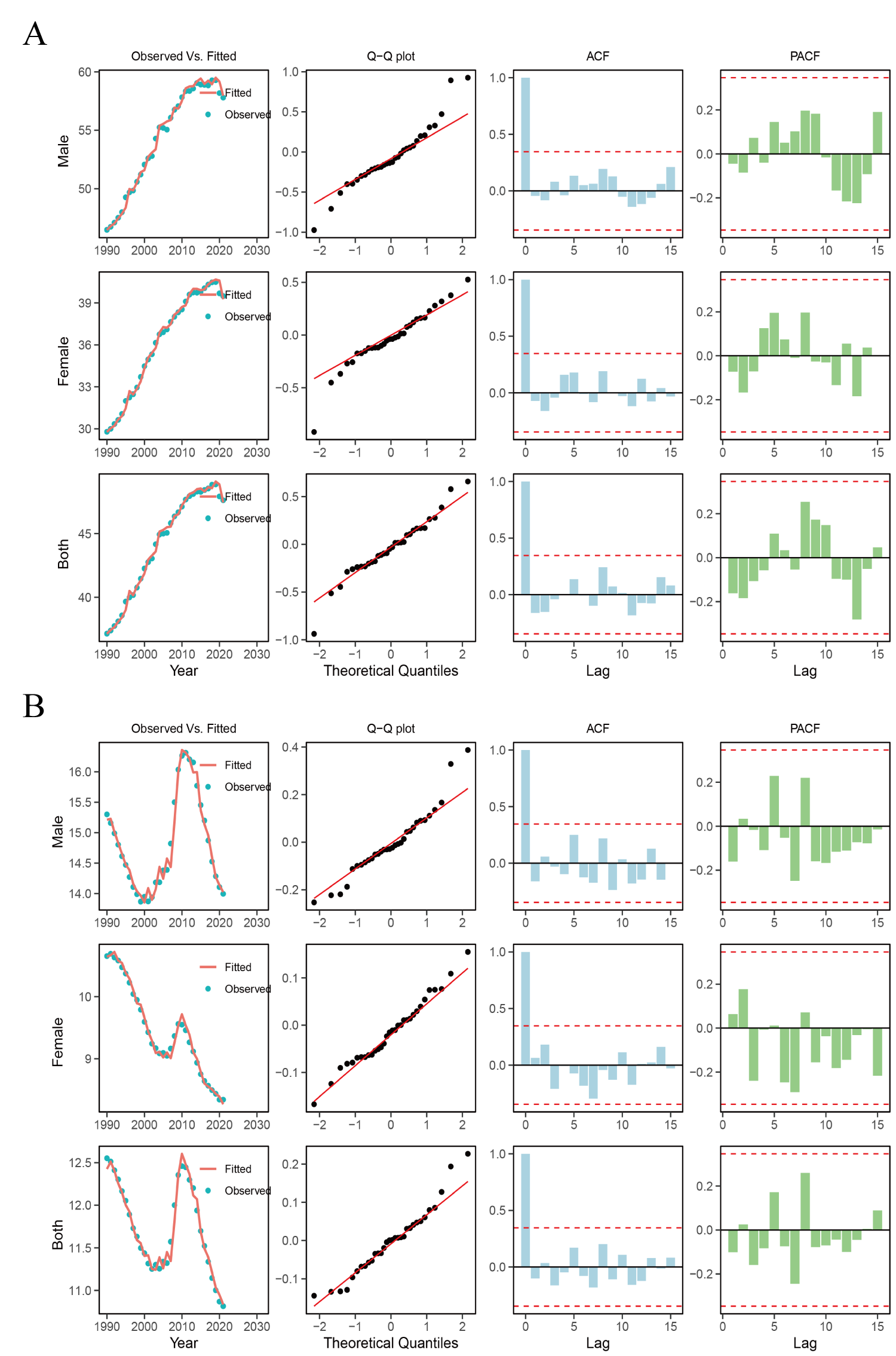


## Figure S1 Comparison of the observed and fitted values of ASDR of LPD in ARIMA models and residual Q-Q plots, autocorrelation function and partial autocorrelation graphs of ARIMA models. (A)Global; (B) China.
